# A tonsil organoid model reveals Epstein–Barr virus–infected germinal center B cell states during primary infection

**DOI:** 10.1073/pnas.2603586123

**Published:** 2026-08-13

**Authors:** Mahina Tabassum Mitul, Yizhe Sun, Timothy B. Yates, Suhas Sureshchandra, Jenna M. Kastenschmidt, Andrew M. Sorn, Zachary W. Wagoner, Erika M. Joloya, Arjun K. Nair, Allyssa Daugherty, Naresha Saligrama, Gurpreet Ahuja, Qiu Zhong, Douglas Trask, Carmy Forney, Rui Guo, Matthew T. Weirauch, Leah C. Kottyan, Benjamin E. Gewurz, Lisa E. Wagar

**Affiliations:** ^a^ Department of Physiology and Biophysics, Institute for Immunology, and Center for Virus Research, University of California Irvine, Irvine, CA 92697; ^b^ Division of Infectious Diseases, Department of Medicine, Brigham and Women’s Hospital, Harvard Medical School, Boston, MA 02115; ^c^Department of Neurology, Washington University School of Medicine in St. Louis, St. Louis, MO 63110; ^d^ Division of Pediatric Otolaryngology, Children’s Hospital of Orange County, Orange, CA 92868; ^e^ Department of Otolaryngology-Head and Neck Surgery, University of California, Irvine, Orange, CA 92868; ^f^Cincinnati Children’s Hospital Medical Center, Cincinnati, OH 45229

**Keywords:** Epstein–Barr virus, tonsil organoids, adaptive immunity, germinal center

## Abstract

Epstein–Barr virus (EBV) colonizes secondary lymphoid tissues to establish persistent infection and is strongly associated with malignancy and autoimmunity. Our understanding of EBV infection biology is hindered by a lack of models that capture infected B cell activity in the lymphoid tissue microenvironment. We therefore developed an EBV human tonsil organoid model to evaluate key B cell states and antiviral responses, including after primary infection. EBV promoted B cell differentiation into germinal center-like phenotypes and transcriptomic analyses highlighted numerous B cell transcriptional programs unique to EBV-infected cells. B cell receptor repertoire analysis revealed that most EBV^+^ B cells underwent class switching but only rarely participated in somatic hypermutation. CD4 T cells, highly activated by organoid infection, limited EBV^+^ B cell outgrowth in both primary and secondary EBV infection. Our findings demonstrate human tonsil organoids as a physiologically relevant model to investigate key aspects of EBV immunity and pathogenesis.

## Significance

Epstein–Barr virus (EBV) is a gamma-herpesvirus that persistently infects nearly all humans and is rarely associated with autoimmunity and cancer. The virus usually infects tonsillar B cells and persists long-term in memory B cells, but the process by which this transition happens remains unclear. Using human tonsil organoids, we show that EBV^+^ B cells acquire unique and surprisingly diverse transcriptional profiles compared to conventional B cells and that CD4 T cells can limit EBV^+^ B cell outgrowth. The uncoupling of canonical B cell processes reveals how EBV co-opts conventional B cell differentiation to support viral persistence. These findings provide insights into primary EBV infection in a physiologically relevant model of the human lymphoid tissue microenvironment.

Epstein–Barr virus (EBV) is a B lymphotropic, gamma-herpesvirus (1). EBV causes a range of B cell lymphomas, T and NK lymphomas, gastric and nasopharyngeal carcinomas, and autoimmune diseases (2–4). Over 95% of adults worldwide are chronically infected (1). Most primary infections are asymptomatic and occur in early childhood. However, when initial infection occurs in adolescence or in early adulthood, a third to a half of individuals develop infectious mononucleosis (IM), a syndrome characterized by massive lymphocyte and natural killer cell activation (5). Waldeyer’s ring, tissue including tonsils, is the primary site of EBV infection, and B cells are the dominant cell target of persistent infection (6). Like other herpesviruses, EBV exhibits a biphasic life cycle, consisting of latent and lytic phases, each defined by distinct viral gene and protein expression patterns (7). After resolution of primary infection, EBV establishes latency in a small pool of memory B cells (8), from which it sporadically reactivates upon plasma cell differentiation to support transmission (9).

There has been great interest and effort to understand EBV’s complex viral life cycle. According to the prevailing “germinal center” model (6), EBV is thought to initially infect naive B cells and trigger their entry into germinal centers (GCs), specialized secondary lymphoid regions of B cell proliferation, somatic hypermutation (SHM), and class-switch recombination (CSR). Infected B cells are posited to undergo sequential changes in viral latency gene expression. EBV latency programs are comprised of one to eight viral protein-encoding genes or noncoding RNAs (ncRNAs) (10–12) and are variably immunogenic depending on the number, type, and functions of the expressed genes. Due to the asymptomatic nature of most primary infections, latency programs have been mostly based on studies of in vitro generated lymphoblastoid cells (LCLs), ex vivo analysis of EBV-induced lymphomas and IM (13, 14) and PCR analyses of bulk peripheral blood or tonsillar cells (6, 15, 16). In vivo, EBV^+^ B cells have been rarely identified in GCs expressing canonical GC markers, including *BCL6* and *AID* (17). Although these infected GC B cells proliferate, they are few in number and display restricted expansion compared to uninfected GC B cells (18). EBV^+^ GC B cells are hypothesized to differentiate into memory B cells, the reservoir for lifelong EBV infection. EBV^+^ memory B cells can be class switched and can show evidence of SHM (19), but EBV^+^ tonsil GC B cells may not participate in the GC response (20). A recent in vitro study using peripheral blood B cells showed that de novo EBV infection induces EBV^+^ B cells transcriptionally resembling GC B cells (21). However, the lack of a tractable human secondary lymphoid model system has left it unclear whether infected B cells actively participate in a GC response during asymptomatic acute or chronic infection.

EBV-associated diseases remain rare, indicating host immunity effectively controls viral pathogenesis in most people. Early viral control is mediated by NK cells and EBV-specific CD4 and CD8 T cells (22–25). EBV-specific memory T cells are polyfunctional and maintain a virus–host balance in the chronically infected host (26, 27). Antigen-specific CD8 T cells expand during IM and are critical for controlling EBV^+^ B cell proliferation, while CD4 T cells clearly limit EBV pathogenesis as underscored by increased EBV-associated lymphoproliferative disease in HIV-infected individuals (28).

Despite numerous efforts, no effective vaccine exists to block primary infection or protect against EBV-associated diseases. Use of animal models to understand EBV infection biology has been a challenge because EBV does not infect mice (29). Although humanized mouse models have been tremendously helpful in understanding the foundational biology of EBV pathogenesis (30, 31), they have limitations related to lymphoid tissue architecture and GC and memory B cell development (30, 32, 33). Due to its asymptomatic nature, it is rarely possible to follow the natural course of the immune response to primary EBV infection in humans in vivo. Tonsil explant models preserve secondary lymphoid tissue architecture and are useful for studying primary EBV infection, including cellular targets and viral gene expression (34). However, explants also present challenges such as reproducibility, generally short longevity, and low throughput. To overcome these challenges and complement existing models, we used a human tonsil organoid model (35–39) to track the early dynamics of EBV infection in tonsillar B cells along with concurrent cellular responses.

## Results

### Establishing and Profiling EBV Infection in Human Tonsil Organoids.

**Fig. 1.**
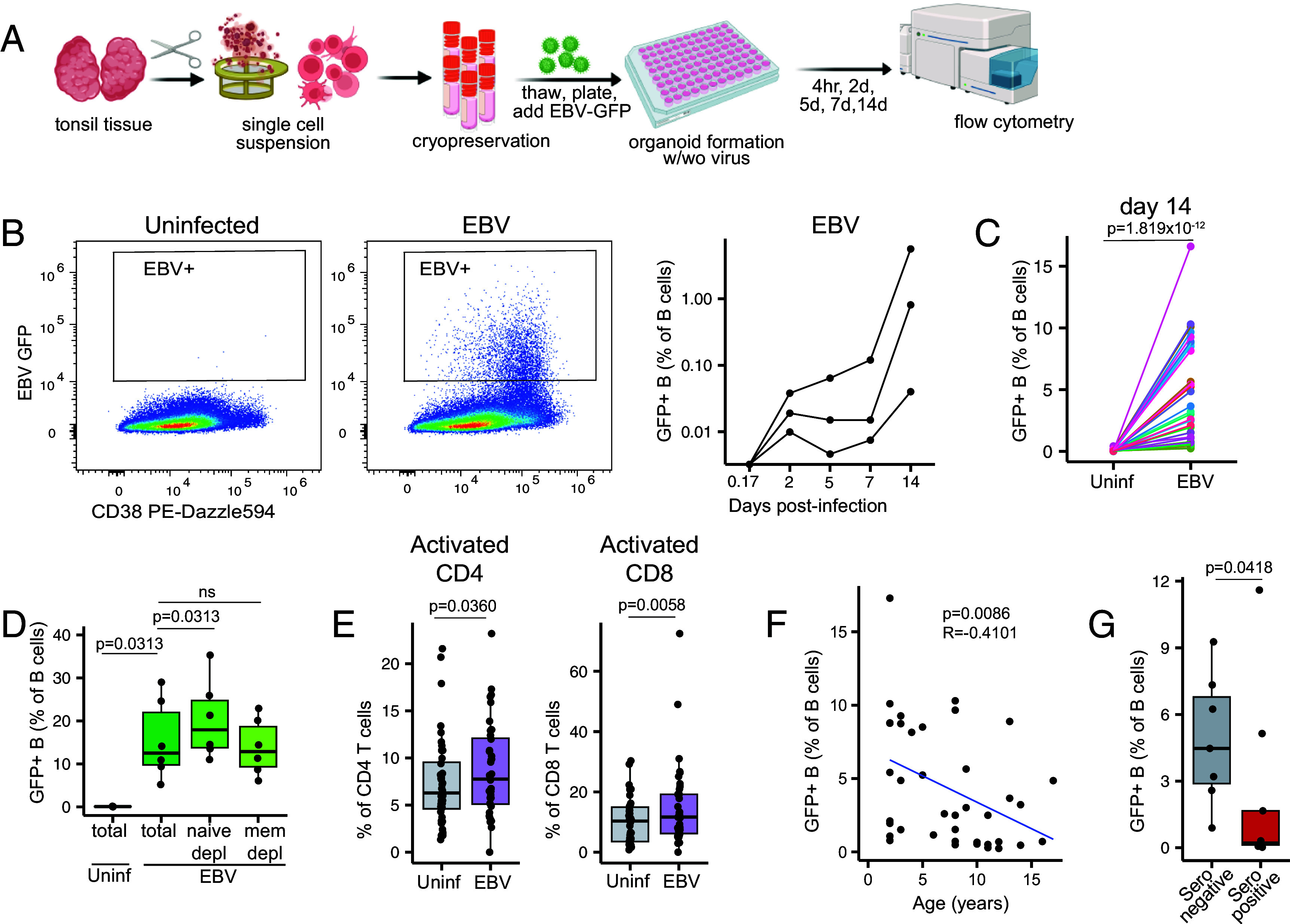
Assessment of EBV^+^ B cells in the tonsil organoids. (*A*) Workflow. Immune organoids were prepared from tonsils and infected with 0.000005, 0.00005, or 0.0005 MOI of GFP-expressing EBV (Akata strain). EBV-infected and uninfected organoids were harvested 4 h, 2 d, 5 d, 7 d, and 14 d post infection and evaluated by flow cytometry. Image created in BioRender. (*B*) Representative flow cytometry plots for EBV^+^ B cells on day 14 and kinetics of EBV^+^ B cells in response to 0.0005 MOI of GFP-expressing EBV (n = 3, each line represents organoids from one donor). (*C*) EBV^+^ B cell frequencies in tonsil organoids on day 14 post infection (n = 40). (*D*) EBV^+^ B cell frequencies on day 14 in uninfected and EBV-infected organoids in the presence or absence of naive (CD38^−^CD27^−^IgD^+^) or memory (CD38^−^CD27^+^IgD^−^ and CD38^−^CD27^−^IgD^−^) B cells (n = 6). (*E*) Activated (CD38^+^HLA-DR^+^) CD4 and CD8 T cell frequencies in the uninfected and EBV-infected organoids on day 14 (n = 40). (*F*) Correlation between tonsil donor age and EBV^+^ B cell frequencies (n = 40). (*G*) Frequency of EBV^+^ B cells on day 14 in EBV-infected tonsil organoids derived from EBV seronegative and seropositive individuals (n = 16; 7 seronegative, 9 seropositive). Mann–Whitney U tests were used to calculate *P* values between groups. Spearman’s rank test was used for correlation analysis. Boxplots indicate the median value, with hinges denoting the first and third quartiles and whiskers denoting the highest and lowest value within 1.5 times the interquartile range of the hinges. MOI, multiplicity of infection; ns, not significant (*P* ≥ 0.05).

Tonsil organoids were generated from tonsillectomy patient samples (*SI Appendix*, Table S1) and infected with the fully transforming, GFP-expressing Akata EBV strain (40), enabling EBV^+^ B cell tracking. EBV titration was performed to establish optimal viral doses (Fig. 1*A* and *SI Appendix*, Fig. S1*A* for representative gating). Consistent with previous EBV studies in tonsil explants (34), EBV-infected GFP^+^ B cells were rare at early time points (median 0 to 0.019% of total B cells from 4 h to 7 d postinfection) but readily detected (median 0.81%) by day 14 (Fig. 1*B* and *SI Appendix*, Fig. S1*B*). While organoids from all donors (n = 40) could be infected, their frequencies varied substantially, demonstrating host heterogeneity in the propensity for EBV infection or expansion of infected cells (Fig. 1*C*).

EBV can immortalize a range of B cells, including naive, unswitched, and switched memory B cells (41–43). Naive B cells are thought to be a major EBV target, whereas resting memory B cells are the persistent reservoir (6, 8). However, it has been difficult to establish which subsets are initially infected and how they differentiate, such as by GC or extrafollicular pathways, prior to entering the memory pool (43–45). To evaluate which B cells are permissive to EBV infection in the tonsil organoid microenvironment, we depleted either memory (CD27^+^CD38^−^IgD^−^ and CD27^−^CD38^−^IgD^−^) or naive (CD27^−^CD38^−^IgD^+^) B cells on day 0, infected with EBV, and later quantified GFP^+^ B cells compared to nondepleted control organoids. Naive B cell depletion led to a significant increase in EBV^+^ B cells, whereas depletion of memory B cells had no impact (Fig. 1*D*). These results indicate that both naive and memory B cells are permissive to EBV infection in a secondary lymphoid microenvironment.

Since T cell responses can restrain EBV^+^ B cell outgrowth, we examined T cell activation (based on CD38 and HLA-DR coexpression) in infected organoids. Total CD4 and CD8 T cell frequencies were unchanged by EBV infection (*SI Appendix*, Fig. S1*C*) but activated phenotypes were modestly but significantly higher in EBV-infected organoids (Fig. 1*E*). We hypothesized that this modest activation may be due to differential availability of viral proteins expressed during latency or lytic replication. To assess whether EBV actively replicates in infected organoids, we transferred culture supernatants from mock infected or day 14 infected organoids onto autologous (uninfected) tonsil organoids or onto Raji B cell targets. Only a small frequency of Raji cells were EBV superinfected, as judged by expression of the EBV-encoded GFP transgene, and no EBV^+^ B cells were detected in organoids on day 7 by flow cytometry, indicating limited replication of the virus in the organoids (*SI Appendix*, Fig. S1*D*). EBV reactivation is restrained by cellular responses in vivo (25, 46, 47). We therefore tested whether donor demographic features such as biological sex, age, and/or EBV seropositivity [as indicated by antibodies against viral capsid antigen (VCA) and Epstein–Barr nuclear antigen (EBNA) in matched serum] were associated with organoid EBV^+^ B cell proportions. While donor sex did not influence the frequency of EBV infection (*SI Appendix*, Fig. S1*E*), organoids from older donors (Fig. 1*F*; *P* = 0.0086) and from EBV seropositive donors had fewer EBV^+^ B cells (Fig. 1*G*; *P* = 0.0418), suggesting that organoids can capture recall responses that limit B cell infection and/or expansion.

### Tonsil Organoid EBV+ B Cells Acquire a GC-like Phenotype.

**Fig. 2.**
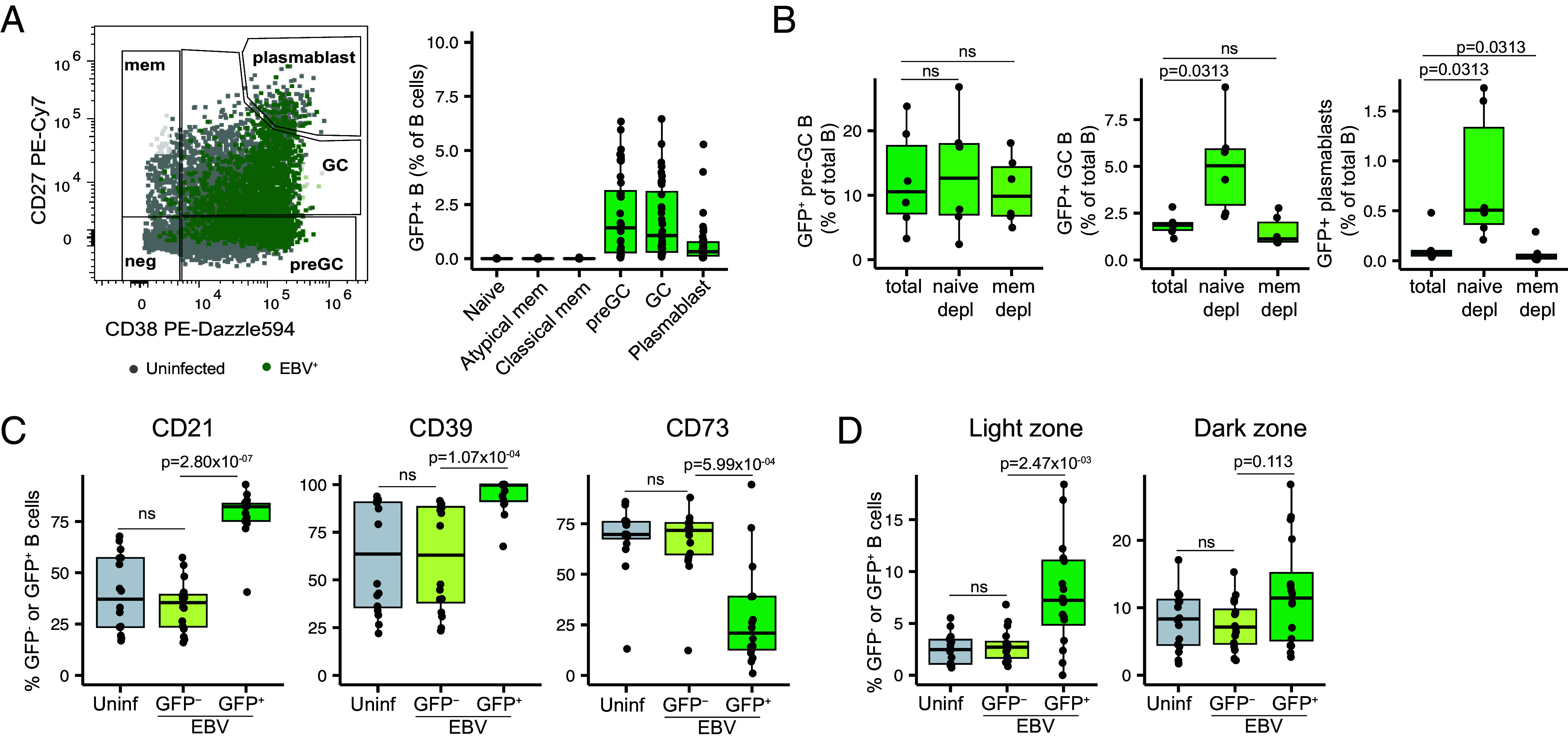
Phenotypes of EBV^+^ B cells in tonsil organoids. (*A*) Representative flow cytometry plot showing EBV^+^ (green) and uninfected (gray) B cells (*Left*) and their frequencies (*Right*) in tonsil organoids on day 14 post infection (n = 40). (*B*) Phenotypes (on day 14) of EBV^+^ B cells when preexisting naive or memory B cells were depleted at the initiation of organoid culture (n = 6). Expression of (*C*) CD21, CD39, CD73, and (*D*) light (CD83^+^) and dark (CXCR4^+^) zone markers in uninfected organoids and GFP^−^ and GFP^+^ B cells from EBV-infected organoids (n = 16). Mann–Whitney U tests were used to calculate *P* values between groups (*B*) and where appropriate, multiple hypothesis correction using the Benjamini & Hochberg method was performed (*C* and *D*). Boxplots indicate the median value, with hinges denoting the first and third quartiles and whiskers denoting the highest and lowest value within 1.5 times the interquartile range of the hinges. ns, not significant (*P* ≥ 0.05).

We next characterized the phenotypes of EBV^+^ B cells over the first 2 wk of culture. Based on CD38, CD27, and IgD patterns, which are commonly used to assign B cell states, most EBV^+^ B cells acquired activated (preGC) or GC-like phenotypes by day 14 (Fig. 2*A*). A few EBV-infected B cells developed plasmablast-like phenotypes. These profiles were specific to tonsil organoids, as parallel organoid experiments using peripheral blood mononuclear cells (PBMCs) or splenocytes as source material showed either inefficient EBV infection (for PBMCs) or fewer pre-GC B cell phenotypes (for splenocytes) compared to tonsils (*SI Appendix*, Fig. S2*A*). Phenotypic profiles of EBV^+^ B cells did not significantly differ between male vs. female donors (*SI Appendix*, Fig. S2*B*), nor were particular phenotypes affected by donor age except for reductions in overall EBV^+^ B cell frequency with increasing donor age (*SI Appendix*, Fig. S2*C*).

Previous work has shown that EBV^+^ B cells can reside within the GC in healthy carriers (17) and in the interfollicular region in IM tonsils (20). To locate EBV^+^ B cells in the tonsil organoids we performed immunofluorescence microscopy. GFP^+^ EBV^+^ B cells were localized predominantly outside of CD10^+^ GC B cell clusters but a few were detected within the CD10^+^ GC B cell compartment (*SI Appendix*, Fig. S2*D*). To further determine whether EBV^+^ B cells remained within B cell follicles, we evaluated CXCR5 expression in EBV^+^ and EBV^−^ B cells in the organoids by flow cytometry. EBV^+^ B cells expressed CXCR5, though to a lesser extent than uninfected B cells, indicating some infected cells may be retained within the B cell follicles (*SI Appendix*, Fig. S2 *E* and *F*). Collectively, these data suggest that EBV^+^ B cells acquire an activated GC-like phenotype and are represented both within and outside follicles, with a subset localizing to the canonical GC in tonsil organoids.

Next, we evaluated the relative contribution of naive vs. memory B cells to the EBV^+^ B cell differentiation phenotypes by individually depleting these subsets prior to establishing organoids. EBV^+^ GC-like and plasmablast B cells were elevated when naive B cells were depleted, while EBV^+^ B cells with a preGC phenotype were unaltered by depletion (Fig. 2*B*). By contrast, organoids established from memory B cell–depleted tonsils exhibited slightly lower levels of EBV^+^ plasmablasts, but pre-GC and GC populations were unaltered. Similarly, depletion of preexisting preGC phenotype B cells largely did not alter the frequency or phenotype of EBV^+^ B cells in tonsil organoids (*SI Appendix*, Fig. S2 *G* and *H*). Together, these data suggest that multiple tonsil B cell subsets can be infected by EBV and they acquire similar phenotypic profiles following infection.

We further characterized other cell surface markers on EBV^+^ B cells that are conventionally used to define lymphoid tissue B cell subsets. Relative to uninfected cells, EBV^+^ B cells were more likely to be CD21^+^, a gp350 attachment receptor for EBV (48), and CD39^+^, an enzyme that hydrolyzes extracellular ATP and ADP (49) (Fig. 2*C*). In contrast, CD73, an enzyme responsible for producing immunosuppressive adenosine (50), was significantly reduced on EBV^+^ cells. Taken together, these results suggest EBV^+^ B cells may modulate extracellular ATP to AMP, potentially for immunomodulation.

The GC is compartmentalized into light and dark zones, where antigen-activated B cells affinity mature, proliferate, and mutate their B cell receptors (BCRs) (44). Although the overall proportions of light and dark zone B cells were unaffected by EBV infection (*SI Appendix*, Fig. S2*I*), EBV^+^ B cells were enriched for expression of the light zone marker CD83 (*P* = 0.00247) but not for dark zone marker CXCR4 (*P* = 0.113) (Fig. 2*D*). Together, these phenotypic differences suggest that early following infection, EBV^+^ B cells have distinct activation, proliferation, and immunosuppressive properties compared to their uninfected counterparts.

### Primary EBV Infection Elicits Unique and Diverse Transcriptional Tonsillar B Cell States.

Although several prospective studies have followed EBV-naive individuals through initial infection (51, 52), primary responses to EBV remain incompletely defined, in part because early infection is often asymptomatic, particularly in young individuals. In symptomatic IM cases, sampling typically happens 4 to 8 wk postinfection (53, 54). Therefore, we leveraged the tonsil organoid model to perform a detailed longitudinal analysis of the early events of primary EBV infection in seronegative donors (*SI Appendix*, Fig. S3*A*). At single cell resolution, we measured gene expression, B and T cell receptor repertoires, and selected protein expression over the first 3 wk of infection in organoids derived from four EBV-naive donors (55). Aggregation of 89,444 cells from all donors, infection conditions, and time points revealed 18 main cell clusters (*SI Appendix*, Fig. S3 *B* and *C* and Dataset S1), which were then subdivided by cell identity for additional analyses.

**Fig. 3.**
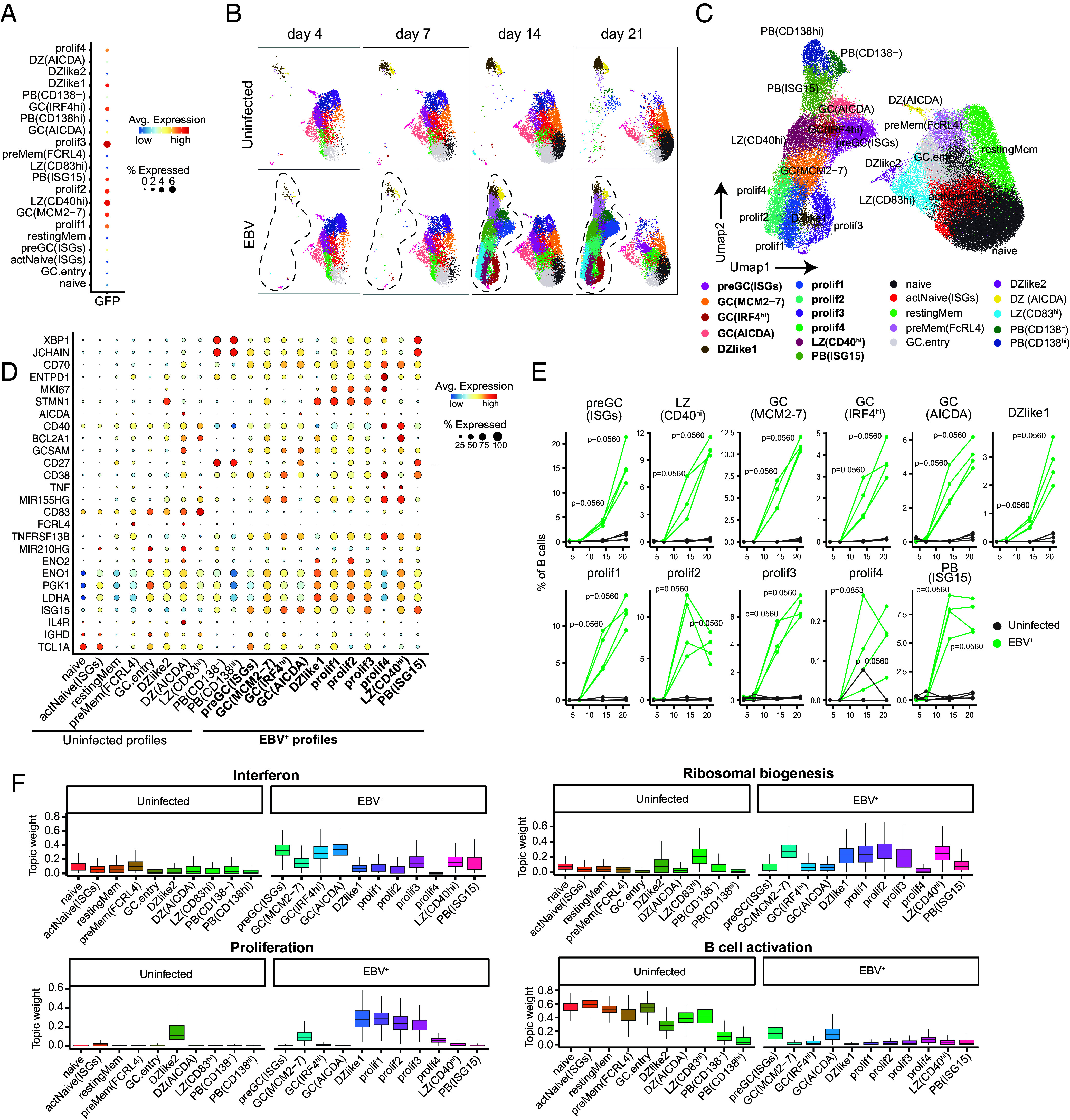
Transcriptional landscape of EBV^+^ B cells in primary infection. (*A*) Detection of GFP transcripts in B cell clusters from EBV-infected organoids. (*B*) UMAP stratification by infection condition and time. Populations enriched following EBV infection are highlighted. (*C*) UMAP of B cells (all donors, timepoints and stimuli combined, 63,263 B cells, n = 4 seronegative donors, 1 experiment). B cell clusters associated with EBV infection in organoids are bolded. (*D*) DEGs used to define B cell cluster identities. (*E*) Frequencies of B cell clusters in uninfected and EBV^+^ organoids (n = 4). (*F*) Topic weights (mean gene expression program) across B cell clusters in EBV-infected organoids. Boxplots indicate the median value, with hinges denoting the first and third quartiles and whiskers denoting the highest and lowest value within 1.5 times the interquartile range of the hinges. Mann–Whitney U tests followed by multiple hypothesis correction using the Benjamini & Hochberg method were used to calculate *P* values.

We first evaluated the B cell transcriptional landscape following EBV infection. Subclustering defined 21 B cell clusters (Dataset S2) and *GFP* mRNA was detected in several of them, indicating EBV^+^ B cells were present across numerous cell states (Fig. 3*A* and *SI Appendix*, Fig. S3*D*). Stratification by organoid infection and time revealed a dramatic, EBV-induced transcriptional change in B cells on days 14 and 21 postinfection (Fig. 3*B*). Further analysis of the top differentially expressed genes (DEGs) at later time points showed remarkable transcriptional diversity in EBV^+^ B cells and almost no transcriptional overlap with uninfected cells (Fig. 3 *C* and *D*; EBV-associated clusters bolded). At a broad level, the 11 EBV-associated clusters spanned GC, plasmablast, and proliferating phenotypes (Fig. 3 *C* and *D* and *SI Appendix*, Fig. S3*E*) and the proportions of these clusters increased over time (Fig. 3*E* and *SI Appendix*, Fig. S3*F*).

Primary B cells derived from peripheral blood have been used previously to understand EBV^+^ B cell phenotypes and viral life cycle. We investigated the transcriptional similarities and dissimilarities between tonsil organoid-derived EBV^+^ B cells and PBMC-derived EBV^+^ B cells at single cell resolution as described in SoRelle et al. (21). Using a quantitative approach where we searched for clusters in the two datasets with at least 10 shared genes in their top DEG profiles, we found a few similar populations (*SI Appendix*, Fig. S3*G* and Dataset S3). Well-aligned clusters included uninfected memory (matched to resting memory), plasmablasts (matched to plasmablasts expressing CD138 and *ISGs*), and proliferating B cells (matched to prolif3) based on transcriptional resemblance (representing 20% overlap). Further, we performed a qualitative comparison based on manual review of top DEGs among the remaining clusters. This qualitative approach revealed partial resemblance to several additional B cell clusters described in SoRelle et al., including c8 naive (matched to naive), c4 antiviral (matched to actNaive-expressing ISGs), c0 preGC (matched to preGC-expressing *ISG*s), and c2 NF-kB (matched to LZ-expressing *CD40*) (*SI Appendix*, Fig. S3*G*). We also noted several unique features of the EBV^+^ populations. Even though many GC-like profiles were present in the EBV^+^ B cell pool, most had low expression of the master transcription factor *BCL6*, as has been noted in previous studies (56, 57). Some of the GC-like, EBV^+^ B cells were primarily defined by conventional GC gene expression features, such as *MCM2-*7 (involved in DNA replication) and *AICDA* (encoding AID, the enzyme that facilitates SHM and class switching). However, the top DEGs for several EBV^+^ GC populations were dominated by interferon (IFN) response signaling, such as IFN-stimulated genes (*ISGs*) and IFN regulatory factors (e.g., *IRF4*) (Fig. 3*D*), demonstrating they were responding to surrounding antiviral signals. Within plasmablasts, an EBV^+^ subset was also strongly defined by *ISG* expression signatures (Fig. 3 *C*–*E* and *SI Appendix*, Fig. S3*E*). Aside from these populations, a substantial proportion of EBV^+^ B cells expressed *MKI67 and STMN1 (*Prolif1-4*)*, indicating a large fraction were proliferating; this proliferation was largely restricted to infected cells. Accumulation of Ki67^+^ CD39^+^ CD70^+^ EBV^+^ B cells has been reported in a humanized mouse model and these EBV^+^ B cells are speculated to proliferate aggressively (58). In tonsil organoids, among the four *MKI67* (encodes Ki67) expressing EBV*^+^* B cell populations, only the Proli4 cluster, and to a lesser extent, Prolif2, coexpressed *CD70* and *ENTPD1* (CD39) (Fig. 3*D*). We did not identify any naive or memory B cell subsets in the EBV^+^ pool (Fig. 3 *C*–*E* and *SI Appendix*, Fig. S3 *E* and *F*), suggesting EBV drove differentiation away from a naive state but not yet to a memory state by day 21 postinfection. Overall, the heterogeneity of GC-like B cell subsets highlights the diversity of EBV-induced B cell transcriptional reprogramming within the secondary lymphoid niche.

By day 14, organoid EBV^+^ B cells exhibited diverse transcriptional states, indicating that the virus exploits multiple functional niches to drive proliferation and survival while bypassing canonical GC checkpoints, thereby supporting viral persistence. We performed topic analysis (59, 60) to summarize key EBV-driven transcriptional remodeling. We identified 15 topics across infected vs. uninfected B cell populations (*SI Appendix*, Fig. S3*H* and Dataset S4). Classification based on top 50 DEGs highlighted IFN response (K6), ribosomal biogenesis (K11), and proliferation (K15) topics in EBV^+^ B cells (Fig. 3*F*). The IFN topic was most evident in GC-like EBV^+^ B cells but also gradually increased in bystander B cells at later time points (*SI Appendix*, Fig. S3*I* and Dataset S5). Ribosomal biogenesis was particularly increased in EBV^+^ proliferating populations (*SI Appendix*, Fig. S3*I* and Dataset S5), indicative of rapid proliferation. Our data reveal that EBV selectively promotes growth in B cells with low expression of the canonical GC regulator *BCL6* in the tonsil microenvironment. This decoupling of proliferation from normal GC checkpoints may point to a mechanism by which EBV expands its reservoir while evading standard GC-mediated regulation.

### A Small Subset of EBV^+^ B Cells Exhibit Conventional GC B Cell Activity.

**Fig. 4.**
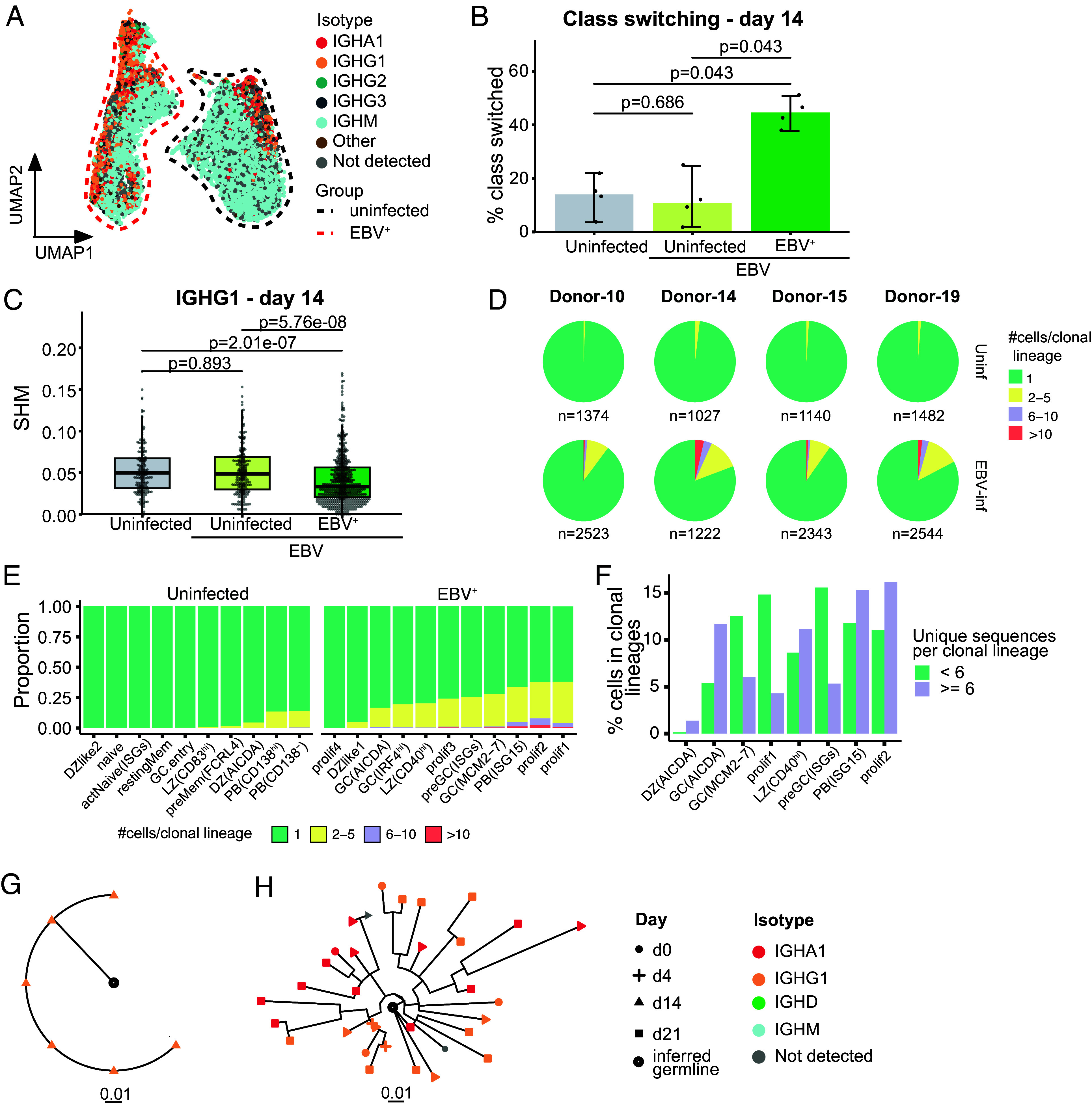
Clonality and isotype usage of EBV^+^ tonsil organoid B cells. (*A*) Antibody isotype usage in EBV^+^ and uninfected B cells from EBV-infected organoids in four donors on day 14. Isotype usage was mapped onto the B cell UMAP generated in Fig. 3. (*B*) Frequency of class-switched isotypes in uninfected, EBV-uninfected, and EBV^+^ B cells on day 14. (*C*) SHM frequency (95% CI) among IGHG1 B cells in uninfected, EBV-uninfected, and EBV^+^ B cells on day 14. (*D*) BCR repertoire diversity in EBV-infected and uninfected organoids from EBV-seronegative donors on day 14. Clonal lineages were defined, then the number of cells per clonal lineage were plotted as a proportion of total B cells. (*E*) Transcriptional profiles associated with clonal expansion. Most expanded clones are in EBV-associated B cell transcriptional profiles. Data from day 14 postinfection. (*F*) Size distribution of clonal lineages containing at least one EBV^+^ B cell. Clonal lineages with more diverse BCR representation (purple) were found primarily in GC-associated cluster phenotypes. Shown are B cell cluster frequencies present in proliferating and GC-like clonal lineages from ex vivo cells and EBV-infected organoids. Lineages (n = 160) with more than 15 cells are included. Examples of clonal lineages in EBV^+^ B cells from (*G*) a simple clonal expansion following infection, and (*H*) an elaborate GC-like clonal lineage with greater BCR diversification, SHM, and class switching. Scale (of 0.01) indicates the branch length representing the number of mutations between each node in the tree. Boxplots indicate the median value, with hinges denoting the first and third quartiles and whiskers denoting the highest and lowest value within 1.5 times the interquartile range of the hinges. Mann–Whitney U tests (two-sided) followed by Benjamini & Hochberg method were used to calculate significance values. SHM, somatic hypermutation; BCR, B cell receptor; CI, confidence interval.

It remains controversial whether EBV^+^ B cells participate in GC reactions, in which B cells proliferate, undergo SHM and CSR, and differentiate into memory or plasmablast phenotypes. While EBV^+^ B cells have been identified in tonsillar GCs (15, 18), they do not show evidence of SHM activity, at least in IM patient tonsils (20). Yet, IM patients have abundant class-switched memory B cells with evidence of SHM (19), suggesting either GC transit, direct memory B cell infection (42), or an EBV-driven mechanism different from conventional B cell differentiation. Recent work indicates EBV^+^ B cells may phenocopy GC dynamics, although they are transcriptionally distinct from conventional tonsillar GC B cells (61). To reconcile these findings and address the plausibility of EBV^+^ B cells participating in an active GC response, we performed BCR and isotype analysis from tonsil organoids. On day 14 postinfection, ~40% of EBV^+^ B cells had class switched, as compared with 10-15% of uninfected B cells from the same organoids and uninfected control organoids (Fig. 4 *A* and *B*). We hypothesized that either class-switched B cells had a higher propensity for infection, or that EBV infection enhanced class switching through an unknown mechanism without increasing SHM. To differentiate between these two possibilities, we looked for evidence of SHM in switched and unswitched B cell populations, since on average ex vivo class-switched B cells would have higher SHM rates than unswitched cells. Analysis of SHM rates by isotype revealed that on average, EBV^+^ cells had similar or even lower SHM rates compared to uninfected B cells of the same isotype (Fig. 4*C* and *SI Appendix*, Fig. S4*A*). The only exception to this was the IgM population, where EBV^+^ cells had slightly higher SHM, though rates were, as expected, very low across all conditions (*SI Appendix*, Fig. S4*A*). These findings are consistent with EBV-induced class switching of naive B cells without, for the most part, induction of SHM in the tonsil organoid microenvironment.

We also analyzed BCR repertoire diversity in EBV^+^ vs. uninfected organoids from the EBV seronegative donors on day 14 postinfection. Consistent with an increased frequency of EBV^+^ GC and proliferating cell profiles, EBV-infected organoids had more B cell clonal expansions and clonal lineages (Fig. 4*D*). Clonal expansions were enriched within EBV^+^ pre-GC, GC, and proliferating clusters (Fig. 4*E*), suggesting that EBV drove proliferation within multiple infected B cell states, including prior to GC entry. The largest and most diverse clonal lineages were observed in EBV^+^ GC-like B cells (Fig. 4*F*). Although 99% (3,408 out of 3,437) of EBV^+^ clonal lineages with at least six B cell members were simple expansions with limited SHM (Fig. 4*G*), we found several examples (n = 29 lineages, 0.84%) of elaborate clonal lineages with SHM (Fig. 4*H* and *SI Appendix*, Fig. S4*B*) that were unique to the EBV^+^ B cell population. Cells from these larger lineages were detected across numerous time points and showed evidence of intralineage class switching, consistent with GC functions (Fig. 4*H*). Based on these findings, we conclude that EBV infection supports isotype class switching and most EBV^+^ B cells undergo small, simple clonal expansions without BCR mutation, but a small subset of infected B cells undergoes SHM in the tonsil organoid microenvironment.

### CD4 T Cells Limit EBV^+^ B Cells in Tonsil Organoids.

**Fig. 5.**
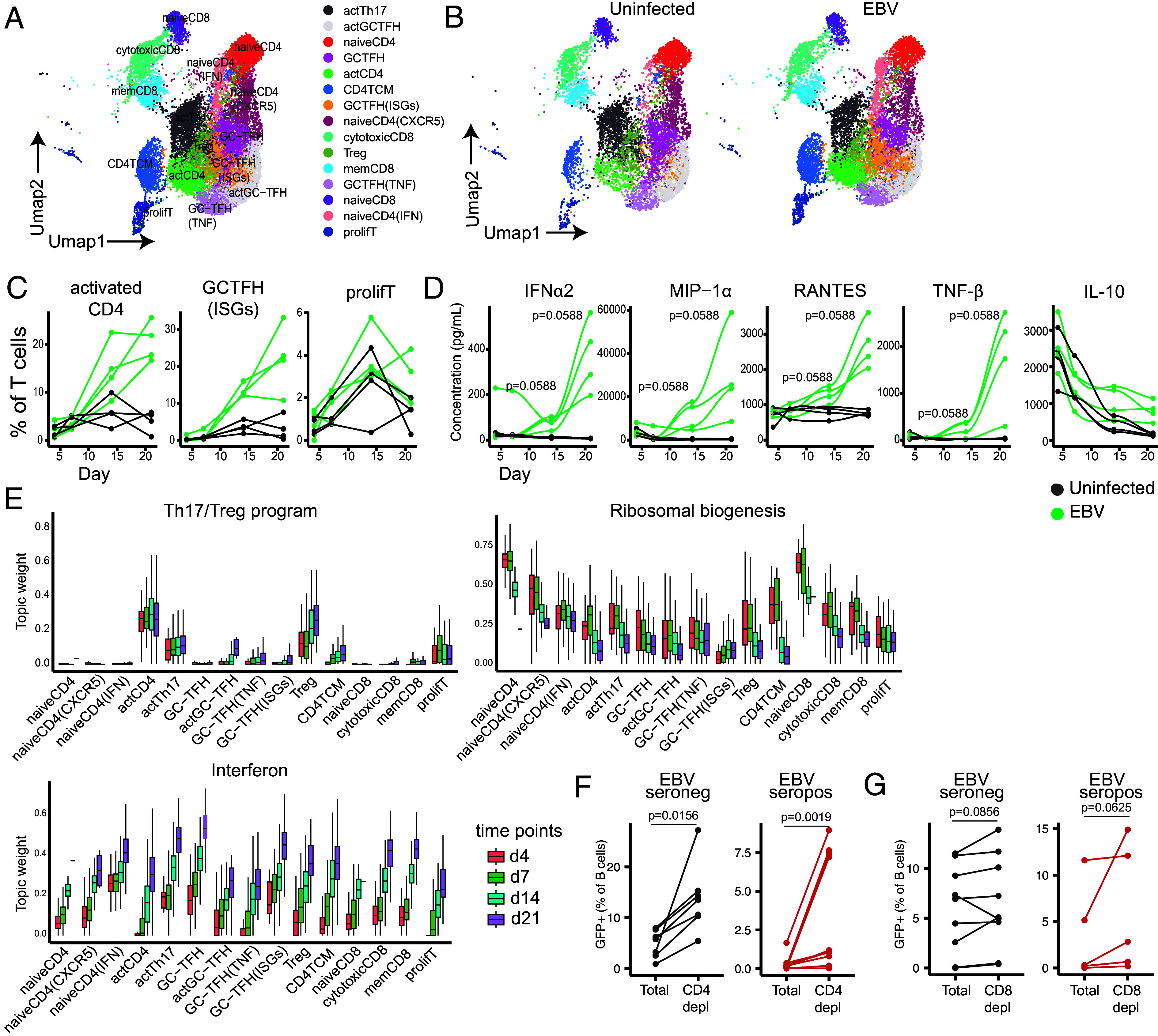
T cell responses to EBV in tonsil organoids. (*A*) Aggregated UMAP representing T cell subsets from EBV seronegative donors (all donors, timepoints and stimulation aggregated; 18,707 cells, n = 4, 1 experiment). (*B*) Distribution of T cell transcriptional profiles in uninfected and EBV-infected organoids. (*C*) Frequencies of activation-associated CD4 T cell clusters over time in EBV-infected (green) and control (gray) organoids (n = 4). No cluster frequencies reached statistical significance between uninfected and EBV-infected organoids. (*D*) Secreted cytokines in uninfected (gray) vs. EBV-infected (green) organoids (n = 4). (*E*) Topic weights (mean gene expression program) in CD4 and CD8 T cell subsets in EBV-infected organoids. EBV^+^ B cell frequencies in organoids on day 14 in the presence or absence of (*F*) CD4 T cells (n = 7 seronegative and n = 10 seropositive donors) or (*G*) CD8 T cells (n = 9 seronegative and n = 5 seropositive donors). Boxplots indicate the median value, with hinges denoting the first and third quartiles and whiskers denoting the highest and lowest value within 1.5 times the interquartile range of the hinges. Mann–Whitney U tests followed by multiple hypothesis correction using the Benjamini & Hochberg method were used to calculate *P* values (*C*–*E*). Mann–Whitney U tests (paired) were used to calculate *P* values between conditions (*F* and *G*).

CD4 and CD8 T cells contribute to EBV control (11, 22, 62, 63), though little is known about initial T cell responses to EBV infection, particularly in the tonsil. A major benefit of the tonsil organoid model is the ability to holistically consider both B and T cells in the same culture environment. Thus, we analyzed CD4 and CD8 T cell transcriptional profiles in the same EBV seronegative donor-derived organoids (*SI Appendix*, Fig. S3*A*). Aggregation and analysis of *CD3^+^* cells (18,707) from all donors, time points, and infection conditions identified 15 T cell clusters (Fig. 5*A* and *SI Appendix*, Fig. S5 *A* and *B* and Dataset S6). Overall, the populations identified were in line with our previous studies of ex vivo and organoid-cultured tonsil T cells (39). In contrast to our B cell analyses, we did not detect any T cell clusters uniquely associated with or induced by EBV (Fig. 5*B*). Rather, the proportion of T cells occupying different clusters shifted over time. By day 14, EBV-infected organoids showed a trend (though not statistically significant due to small sample size) for increased proportions of activated CD4 T cells and germinal center T follicular helper (GC-TFH) cells with *ISG* expression profiles, coinciding with EBV^+^ B cell outgrowth (Fig. 5*C*). Minor increases in these populations, in addition to a proliferating T cell population, started on day 4 and peaked on day 14 postinfection (Fig. 5*C*). Other T cell populations, including memory CD8 T cell subsets conventionally associated with EBV infection in peripheral blood studies, were largely unchanged following tonsil organoid infection (*SI Appendix*, Fig. S5*C*). Moreover, none of these CD4 and CD8 T cell subsets in EBV-infected organoids showed exhausted profiles (*SI Appendix*, Fig. S5*A*) as recently described in case of CD8 T cells in IM tonsils (64). This disparity may be due to the exceptionally low EBV inoculum used to infect organoids and the kinetics and pattern of EBV gene expression in a naive response. As a complementary analysis, secreted cytokines were quantified from organoid supernatants. Among the 36 cytokines quantified, IFN2α, MIP-1α, RANTES, TNF-β were significantly elevated in EBV-infected organoids (Fig. 5*D*). IL-10 is an important cytokine in EBV infection, and the virus encodes a homolog to modulate IL-10 signaling; we saw a small, but not significant increase in IL-10 levels following EBV infection. Overall, both proinflammatory and anti-inflammatory cytokines were induced in organoids in response to EBV.

Topic modeling was performed to further characterize changes in T cell states following EBV infection. Eight topics were identified across the T cell dataset (*SI Appendix*, Fig. S5*D* and Dataset S7). Annotated topics of interest included a Th17/Treg program (k1), protein synthesis/ribosomal biogenesis (k2), cytotoxicity (k3), proliferation (k4), interferon response (k6), and hypoxia/apoptosis (k7). Most topics corresponded well with our prior T cell annotations based on top DEGs except for the Th17/Treg topic (k1), which was significantly enriched in activated CD4, Th17, and Treg clusters in infected compared to uninfected organoids (Fig. 5*E* and Dataset S8). CD4 T cell polyfunctionality has been described in response to EBV; it is plausible that these CD4 T cell subsets exhibit concurrent pro- and anti-inflammatory features. We observed significant induction of the IFN-related topic across all T cell subsets in infected organoids (Fig. 5*E* and Dataset S8), suggesting a strong and early antiviral response. In contrast to B cells, the ribosomal biogenesis topic was significantly downregulated across many CD4 and CD8 T cell populations following infection (Fig. 5*E*), perhaps due to IFN-induced antiviral mechanisms. We assessed whether EBV infection perturbed T cell repertoire diversity and found there were no substantial changes in CD4 or CD8 T cell clonality (*SI Appendix*, Fig. S5*E*). T cells likely have distinct functions in acute vs. chronic phases of EBV infection and it is possible that clonal expansions would be observed beyond day 21. Finally, we tested the ability of CD4 vs. CD8 T cells to control EBV infection through depletion studies in a separate cohort of tonsillectomy samples from EBV seropositive and EBV seronegative donors. CD4 T cell depletion led to a significant increase in EBV^+^ B cells in both EBV seropositive and seronegative donors (Fig. 5*F*). In contrast, CD8 T cell depletion did not substantially change EBV^+^ B cell proportions in tonsil organoids derived from EBV seropositive or seronegative donors (Fig. 5*G*). Overall, these results highlight a major role for CD4 T cells in controlling EBV^+^ B cell outgrowth in the tonsil organoid microenvironment during both primary and secondary infection.

## Discussion

**Fig. 6.**
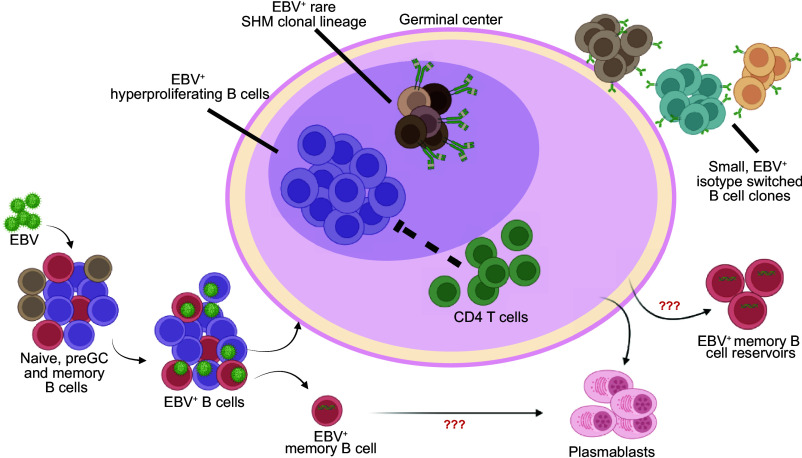
A working model of primary EBV infection in human tonsil organoids. Upon infection, B cells acquire diverse phenotypes, some of which are GC-like. Most infected cells, located outside the canonical GC, class switch and undergo modest proliferation without BCR mutation, leading to many small clonal expansions. A tiny fraction of EBV^+^ B cells reside inside the GC and engage in SHM and develop elaborate clonal lineages. Both populations can differentiate into EBV^+^ plasmablasts although the differentiation path to long-term resting memory B cells remains unclear. CD4 T cells inhibit EBV^+^ B cell outgrowth. Image created in BioRender.

EBV is a strictly human pathogen and while major progress has been made, aspects of acute and chronic infection remain difficult to model in animal models. To complement these efforts, human tonsil organoids were used to investigate primary EBV infection. EBV^+^ B cells were phenotypically and transcriptionally distinct from uninfected cells, including numerous and diverse cell states not previously reported. EBV^+^ B cells exhibited a greater propensity to class switch but had comparatively low SHM rates. Although many EBV^+^ cells showed signs of proliferation and underwent small clonal expansions, only a tiny fraction of B cell clones showed SHM patterns consistent with genuine GC participation. Along with B cell studies, we defined features of the early T cell response to EBV infection and found a critical role of CD4 T cells in controlling EBV infection. Based on these findings, we present a working model of the dynamics of initial EBV infection in the tonsil (Fig. 6) and our data suggest tonsil organoids are a physiologically relevant model for studying EBV biology in a human tissue context.

The GC model of EBV infection proposes that in vivo, EBV^+^ naive B cells transit through the GC to establish latency in the memory B cell pool. In support of this hypothesis, EBV^+^ B cells have been identified in the GC and within interfollicular regions of latently infected tonsils (17), where they are posited to undergo varying degrees of proliferation balanced by cell death. More recently, they have also been identified in the marginal zone (65). Our immunofluorescence imaging data indicate that most EBV^+^ B cells reside outside a canonical GC, yet many continue to express CXCR5, suggesting some retention in B cell follicles. Furthermore, though the majority of organoid EBV^+^ B cells reside outside the GC, they also lacked expression of extrafollicular atypical B cell markers such as *CD11C*, *TBX21*, *ZEB2*, and *ITGAX* (66–69). The localization of EBV^+^ B cells may be influenced by the dynamics of the infection, such as disruption of the GC during IM (20). However, whether the EBV^+^ B cells participate in a bona fide GC reaction has been uncertain (18, 20), particularly in the setting of acute infection. We now provide experimental evidence that EBV^+^ B cells can indeed participate in a genuine GC reaction in a secondary lymphoid model. This was underscored by the identification of a small subset of EBV^+^ B cells with extensive SHM and clonal expansion, highly suggestive of transit through a genuine GC. Such sporadic participation of the EBV^+^ B cells in a true GC reaction offers experimental support for the EBV GC model. Also consistent with the rarity of this subset, a recent study presents an updated GC model where extrafollicular B cells can contribute during EBV infection (65).

We found that EBV could directly infect a variety of B cell subpopulations, including memory cells. Notably, a tonsil explant EBV infection model showed more EBV DNA in memory B cells compared to naive B cells at 24 d postinfection, consistent with memory B cells as a major reservoir of EBV latency (8, 34). Furthermore, EBV is detectable in peripheral blood unswitched memory B cells in both acute and chronic infection, possibly indicating direct EBV infection of extrafollicular memory cells (42). In our model, the enhanced infectivity of memory B cells in the absence of naive B cells might be due to differences in the relative abundance of total naive vs. memory B cells in ex vivo tonsils. Although “atypical” B cells have been extensively described in chronic infection and inflammatory conditions (70, 71), we did not detect EBV^+^ B cells with atypical memory B cell phenotypes by flow cytometry or by transcriptional profiling. One explanation for the overall absence of these cells may be the young age range of the tissue donors used in the experiments. A less diversified antigen exposure history and relatively immature immune landscape in young children may limit the accumulation and differentiation of atypical B cells, which are likely driven by cumulative immune activation, repeated viral exposure, and chronic inflammation in aging. Indeed, compared to classical memory B cells, the ex vivo frequencies of atypical memory B cells in the pediatric population is very limited (37).

At the transcriptional level, EBV^+^ B cells were heterogeneous and distinct from uninfected cells. In our model, the majority of the EBV^+^ B cells resided outside the GC but lacked expression of extrafollicular atypical B cell markers (66–69). Moreover, EBV^+^ B cells subsets were transcriptionally distinct from those recently identified in IM tonsils (64) as well as those from EBV-infected PBMCs (21). We believe there are likely several reasons for these differences. Our data model the first 21 d of seronegative donor primary infection, whereas clinical mononucleosis symptoms present approximately 4 to 8 wk postinfection. EBV lytic replication is a common feature of IM, whereas tonsil organoids had limited lytic replication over the time points studied. GC B cells are largely restricted to lymphoid tissues and their phenotypes, transcriptional and functional features may not be well represented in PBMC-derived B cells. However, differences in virus strain, MOI, clustering resolution, and sequencing depth can further complicate direct comparison. Newly EBV-infected peripheral blood B cells (72) and LCLs (73, 74) show upregulation of both CD39 and CD70. Furthermore, DLBCL-like CD39^+^ CD70^+^ Ki67^+^ EBV^+^ B cells are proposed to have high proliferative potential in a humanized mouse model of EBV (58). Among the four proliferating EBV^+^ B cell clusters in organoids, only some coexpressed these markers, again reinforcing greater heterogeneity of EBV^+^ B cells in tonsil organoids compared to those described in animal models or other in vitro platforms.

Additionally, the diversity of the EBV^+^ cells likely reflects viral manipulation of GC-associated programs to accommodate immune pressure while promoting infected cell survival. Notably, EBV-induced GC-like B cell subsets expressed low levels of *BCL6*, the master regulator of GC B cells, consistent with prior reports demonstrating *EBNA3C* and EBV miRNA-mediated suppression of BCL6 (56, 57, 75, 76). Within the GC, the light zone mediates selection and affinity maturation. Flow cytometric analysis revealed that EBV^+^ GC B cells were enriched for a light zone phenotype, while dark zone frequencies were comparable between EBV^+^ and uninfected populations. EBV latent membrane proteins LMP1 and LMP2A mimic CD40 and BCR signaling, respectively, potentially enabling EBV^+^ B cells to persist in a light zone-like state independent of high-affinity antigen (77–79). Accordingly, enrichment of light zone-like cells among EBV^+^ B cells suggests a preferential survival strategy, potentially involving enhancer-mediated upregulation of *BCL2A1* (80). In contrast, the unchanged frequency of dark zone B cells supports EBV-mediated subversion of the conventional affinity maturation cycle, consistent with our BCR sequencing data showing CSR in the absence of SHM for most infected B cells.

Although EBV can upregulate AID, the enzyme that mediates SHM and CSR (72, 81), these processes are mechanistically and spatially separable. *AICDA* mRNA was detected in one EBV^+^ GC-like subset. Yet, most EBV^+^ B cells class switched without undergoing SHM. This is consistent with limited data from IM patients, which found that EBV^+^ B cells have higher levels of CSR than uninfected B cells (82). Notably, CSR can be induced by strong CD40 signaling or by its EBV mimic LMP1, which can promote extrafollicular CSR independent of cognate T cell help (83–86). Such uncoupling of CSR from SHM may favor EBV persistence by limiting potentially deleterious mutagenesis of the double-stranded DNA viral genome, which could otherwise be a consequence of extensive SHM. Along with CD40 signaling, cytokines including IL-10, IL-4, and TGF-β are known regulators of CSR (87). In addition to inducing host IL-10 expression, the EBV encoded IL-10 homolog BCRF1 (88) may promote CSR, perhaps through leaky lytic gene expression. The relative contributions of host-derived cytokines and viral cytokine homologs to EBV-driven CSR remain to be determined and warrant further investigation.

In contrast to a tonsil explant model (34), we detected limited viral replication in organoids. CD4 and CD8 T cells exert anti-EBV responses to both latent and lytic antigens (25). CD4 T cells specific for peptides from EBV latency proteins exhibit Th1-like profiles but without overt expansion of the CD4 T cell compartment (89). EBV-specific CD8 T cells often target lytic antigens in early IM (22, 90) and in IM patients, CD8 T cells specific for EBV lytic and latent peptides can represent 1 to 40% and 0.1 to 5% of total circulating CD8 T cells, respectively (24, 91, 92). Activated CD4 and CD8 T cell frequencies each increased in response to EBV in the tonsil organoids, though we observed relatively stable CD4 and CD8 T cell repertoire diversity despite CD4 T cell transcriptional activation and T cell–derived cytokine production. The limited CD8 T cell activation may thus reflect the extremely low viral dose used to infect the organoids, low lytic replication, as well as the young age of the EBV-seronegative tonsil donors (<13 y), in whom primary EBV infection is often clinically mild or asymptomatic.

Despite a largely stable CD4 T cell repertoire, we observed a critical role for CD4 T cells over CD8 T cells in controlling primary and secondary EBV infection in tonsil organoids. In the absence of CD4 T cells, the frequency of EBV^+^ B cells increased in organoids generated from seropositive and negative donors, indicating that both memory CD4 T cells and newly activated or bystander CD4 T cells control EBV^+^ B cell expansion. The limited CD8 T cell role might be due to limited frequencies or functions of EBV-specific CD8 T cells in the tonsil in acute infection. CD8 T cells are enriched in IM patient tonsils but these cells exhibit limited functional capacity, ultimately leading to incomplete viral control (64), even though high frequencies of EBV-specific tonsillar CD8 T cells are observed in long-term EBV carriers (93). Consistent with our findings, a humanized mouse model of EBV infection showed tissue-resident memory CD8 T cell expansion at the site of infection, but they failed to control local EBV viral load despite retention of cytotoxic function (94). In children and adults with primary EBV infection, granzyme B and perforin-secreting CD4 T cells have been identified in tonsils and peripheral blood, respectively (47, 95) and the cytotoxic profile of Th1-like CD4 cells is retained in the tonsil throughout the lifetime (96). LMP1 signaling induces potent T cell responses, including cytotoxic CD4 T cells (62, 86). Polyfunctional T cells may help to control chronic infection (97), and they are more abundant in healthy carriers than in acute IM patients, predominantly expressing TNFα and IFNγ (47). Consistent with this, the cytokine response during organoid primary EBV infection was dominated by type I IFN, TNFα, RANTES, and TNFβ, but without significant changes in IFNγ, suggesting a microenvironment resembling asymptomatic infection. Nonetheless, the tonsil organoid model offers further opportunities to study anti-EBV T cell responses in primary vs. secondary infection, including mechanisms of EBV immune evasion that can be challenging to assess in explant models (34) or other in vitro platforms.

Although the tonsil organoid model provides an excellent platform to evaluate infection and tissue immunity to different viral and vaccine antigens, in the context of EBV infection the current study has a few limitations. While EBV^+^ B cell differentiation into memory cells has been observed in vitro (21), we did not identify EBV^+^ memory B cells in the organoids. It is possible that the 3-wk culture period was not sufficient to establish a pool of EBV^+^ memory B cells. Alternatively, EBV^+^ memory B cells may have silenced all EBV gene expression and become indistinguishable from conventional, uninfected memory B cells in the organoids. Future studies will delineate the temporal dynamics of EBV gene expression in the tonsil organoid system to associate viral gene expression patterns with host B cell transcriptional states during infection. Moreover, the tonsil epithelium is not well represented in organoids and major antigen-presenting cell populations that would be replenished in vivo from migration into and out of the tonsil from the periphery are underrepresented. Thus, the absence of the epithelium and these innate immune cells prevented us from evaluating the relative contributions of these cells to EBV infection and immune responses to EBV.

In summary, the tonsil organoid model provides a highly tractable platform to investigate EBV infection-mediated changes in B cell biology and concurrent T cell responses. The presence of infected and uninfected B cell subsets in the same microenvironment also highlights B cell heterogeneity during initial EBV infection as well as the distinction between tonsil organoids and other contemporary in vitro platforms. Simultaneous assessment of cellular immune responses to EBV can also readily be modeled in organoids established from seronegative or seropositive donors. The EBV tonsil organoid model offers a versatile platform to investigate EBV pathogenesis, antiviral responses, and therapeutic strategies not accessible in conventional animal models or standard in vitro systems.

## Methods

### Tissue Processing, Organoid Preparation, and Infection with EBV.

Tonsils: Tonsil pairs from healthy individuals were collected from the Cooperative Human Tissue Network (CHTN) at the Vanderbilt Medical Center (VUMC), the University of California Irvine Medical Center (UCIMC) or from Children’s Hospital of Orange County (CHOC), from Washington University in St. Louis (WUSTL), or from Cincinnati Children’s Hospital (CCHMC). Patients underwent tonsillectomy for obstructive sleep apnea or hypertrophy and tonsil tissues were healthy in appearance. CHTN samples were collected as discarded surgical materials (deemed not human subjects research by the UCI IRB) and provided as deidentified samples. Participants at UCIMC/CHOC, WUSTL, and CCHMC provided written, informed consent, and, where applicable, assent through institutionally approved IRB protocols that covered both specimen collection and research use of the tissues for the types of analyses reported here (UCI IRB 2020-6075, WUSTL IRB 202103116, CCHMC IRB 2018-3069). For immunohistochemical analyses, tonsils from healthy individuals were also collected from CCHMC and analyzed under CCHMC protocol 2022p001286. All tissue samples were deidentified and assigned unique alphanumeric codes in accordance with HIPAA regulations and institutional guidelines.

#### Spleens.

Spleen samples (n = 4) were collected from patients undergoing splenectomy at UCIMC and CHOC as surgical discard material and provided to the lab as deidentified tissue samples through an honest broker system with UCI Pathology.

#### Blood samples.

For tonsil donors consented through UCI/CHOC and WUSTL, a subset of participants also agree to provide a blood sample as part of the informed consent process and approved protocols as above. Whole blood was collected at the time of the patients’ surgeries. Deidentified whole blood (n = 5) was also received from UCI Institute for Clinical and Translational Science (ICTS) blood donor program under approved UCI IRB protocol #2001-2058.

Tonsil pairs were processed as previously described (35). In brief, the tonsils were mechanically dissociated in Ham’s F12 media with 2% FBS in a metal processing cup. The cell suspensions were then filtered through a 100 μm nylon strainer and subjected to density gradient centrifugation. The purified live tonsillar cells were enumerated and cryopreserved in FBS with 10% DMSO at −140 °C until use. The spleen tissues were processed on the same day postsurgery following the procedure described above. Serum and PBMCs were separated from whole blood samples by centrifuging the whole blood at 1,000×*g* for 5 min at room temperature. The serum and PBMC samples were stored at −80 °C and −140 °C, respectively, until used. The demographic information of all the tissue donors is provided in *SI Appendix*, Table S1.

To generate the tonsil organoids, cryopreserved aliquots were thawed, enumerated, and plated in transwells or ultra-low attachment (ULA) 96 well flat bottom plates. The final density of cells was 12 × 10^6^ cell/mL in a final volume of 500 μL (6 × 10^6^ cells per well) in the transwells or 7.5 × 10^6^ cells/mL in a final volume of 200 μL (1.5 × 10^6^ cells per well) in the 96 well ULA plates. Spleen organoids and PMBCs were plated in the 96 well plates having 7.5 × 10^6^ cells/mL in a final volume of 200 μL (1.5 × 10^6^ cells per well). Following plating tonsil cells, splenocytes, or PBMCs, GFP-expressing EBV was added in respective wells to generate EBV-infected organoids. The organoid media was composed of RPMI1640 with glutamax, 10% FBS, 1× nonessential amino acids, 1× sodium pyruvate, 1× penicillin/streptomycin, 1× Normocin, 1× insulin/selenium/transferrin cocktail, and 1 μg/mL of human BAFF (produced recombinantly and purified in-house or from Biolegend or SinoBiological). Additional experimental details are provided in *SI Appendix*.

## Supplementary Material

Appendix 01 (PDF)

Dataset S01 (XLSX)

Dataset S02 (XLSX)

Dataset S03 (XLSX)

Dataset S04 (XLSX)

Dataset S05 (XLSX)

Dataset S06 (XLSX)

Dataset S07 (XLSX)

Dataset S08 (XLSX)

## Data Availability

Single-cell RNA sequencing data have been deposited in Gene Expression Omnibus database (GSE317492; https://www.ncbi.nlm.nih.gov/geo/) (55). All other data are included in the manuscript and/or supporting information.
