# Supplementary material for: A tonsil organoid model reveals Epstein–Barr virus–infected germinal center B cell states during primary infection
Source: Proc Natl Acad Sci U S A. 2026 Aug 13;123(33):e2603586123. doi: 10.1073/pnas.2603586123 (PMC13486580; doi:10.1073/pnas.2603586123)
Supplement: Supplementary file 1 [file pnas.2603586123.sapp.pdf]

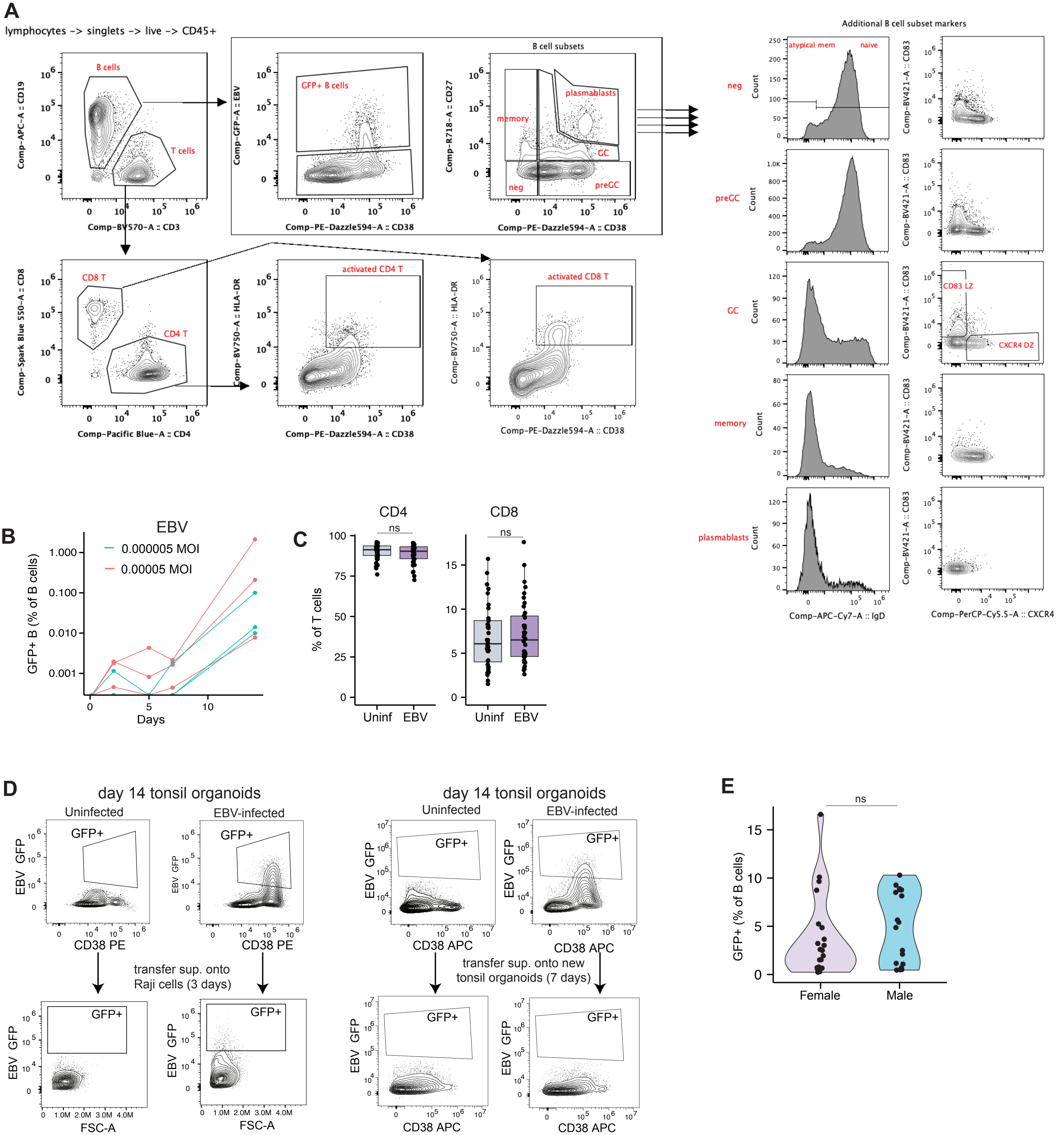

Figure S1: Extended analysis of B and T cell responses in tonsil organoids following EBV infection. (A) Gating strategy: Representative gating strategy for identifying B and T cell subsets in tonsil organoids. (B) Frequencies of EBV$^+$ B cells in response to 0.000005 and 0.00005 MOI of GFP-expressing EBV (n=3, each line represents organoids from one donor). (C) Frequencies of total CD4 and CD8 T cells in uninfected and EBV-infected tonsil organoids on day 14 (n=40 per group). (D) Flow cytometry plots showing EBV$^+$ Raji cells on day 3 (bottom left panel) or EBV$^+$ B cells on day 7 (bottom right panel) in autologous organoids when day 14 culture supernatants from uninfected or EBV-infected organoids were used to infect the indicated target cells (Raji or organoids). (E) Frequencies of EBV$^+$ B cells in organoids derived from female and male tonsil tissue donors (n=40, 21 female, 19 male). Mann Whitney U tests were used to calculate p values between groups. Boxplots indicate the median value, with hinges denoting the first and third quartiles and whiskers denoting the highest and lowest value within 1.5 times the interquartile range of the hinges. MOI, multiplicity of infection; ns, not significant ($p \geq 0.05$).

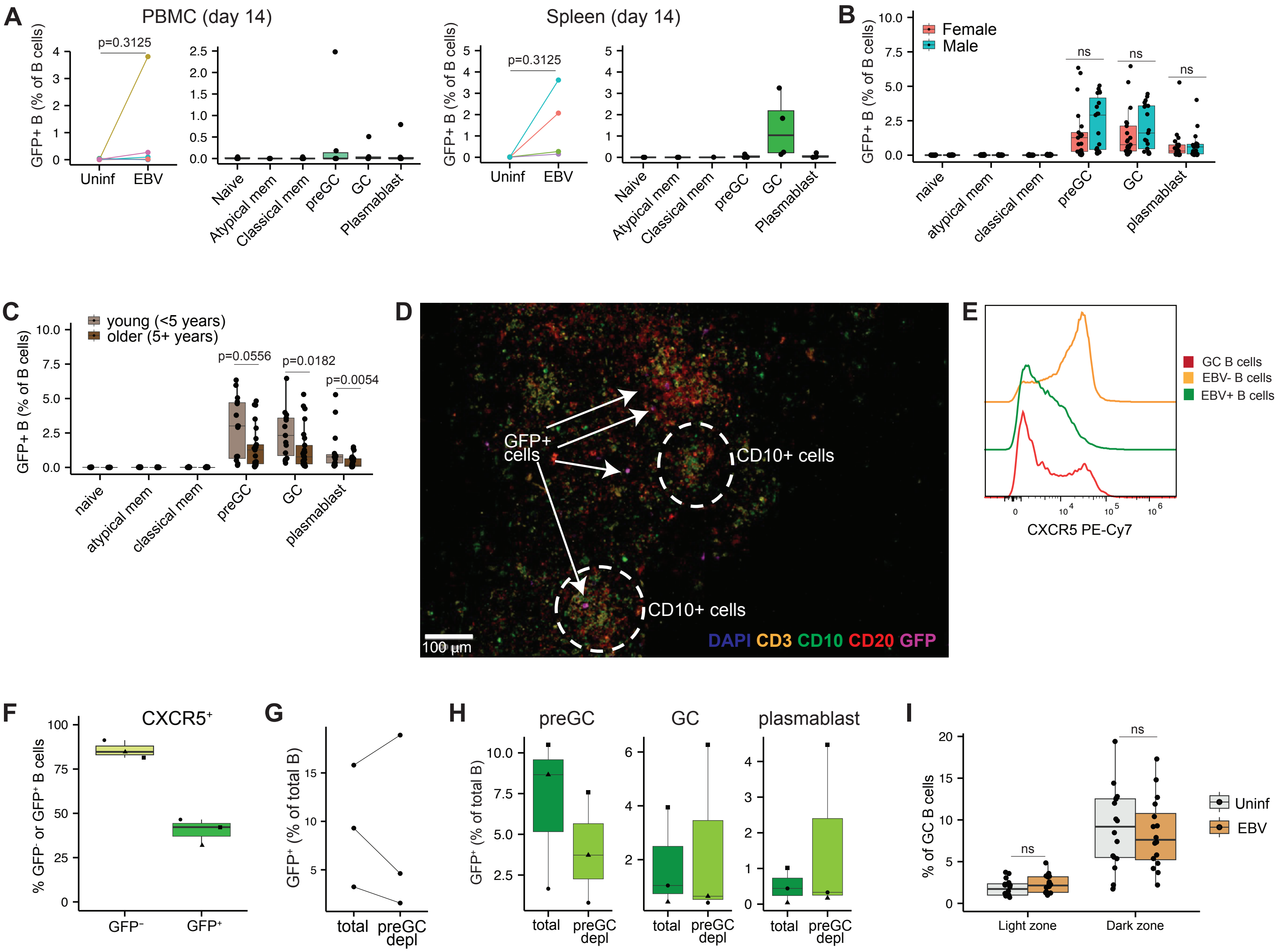

Figure S2: Additional phenotypic differences between EBV+ and EBV- B cells. (A) EBV+ B cell frequencies and phenotypes in uninfected and EBV-infected PBMCs (n=6 per group) and spleen organoids (n=4 per group). (B) EBV+ B cell phenotypes in organoids derived from male and female tissue donors (n=40, 21 female, 19 male). (C) EBV+ B cell subsets in younger (<5 years old, n=15) and older (5+ years old, n=25) children. (D) Immunofluorescence microscopy from EBV-infected organoids showing the location of GC B cells (CD20+ CD10+) and GFP+ cells. Scale bar, 100 µm. (E) Representative histogram showing CXCR5 expression levels in total GC B cells (red), uninfected B cells (orange), and EBV-infected B cells (green) from EBV-infected organoids. (F) Frequencies of GFP+ and GFP- B cells expressing CXCR5 in EBV-infected organoids (n=3). Frequencies of (G) EBV+ B cells and (H) EBV+ B cell phenotypes in the presence and absence of pre-existing preGC B cells (n=3). (I) Light zone (CD83+) and dark zone (CXCR4+) GC B cell frequencies in uninfected and EBV-infected tonsil organoids (n=16). All data shown are derived from day 14 cultures. Mann Whitney U tests were used to calculate p values between groups. Boxplots indicate the median value, with hinges denoting the first and third quartiles and whiskers denoting the highest and lowest value within 1.5 times the interquartile range of the hinges. ns, not significant ($p \geq 0.05$).

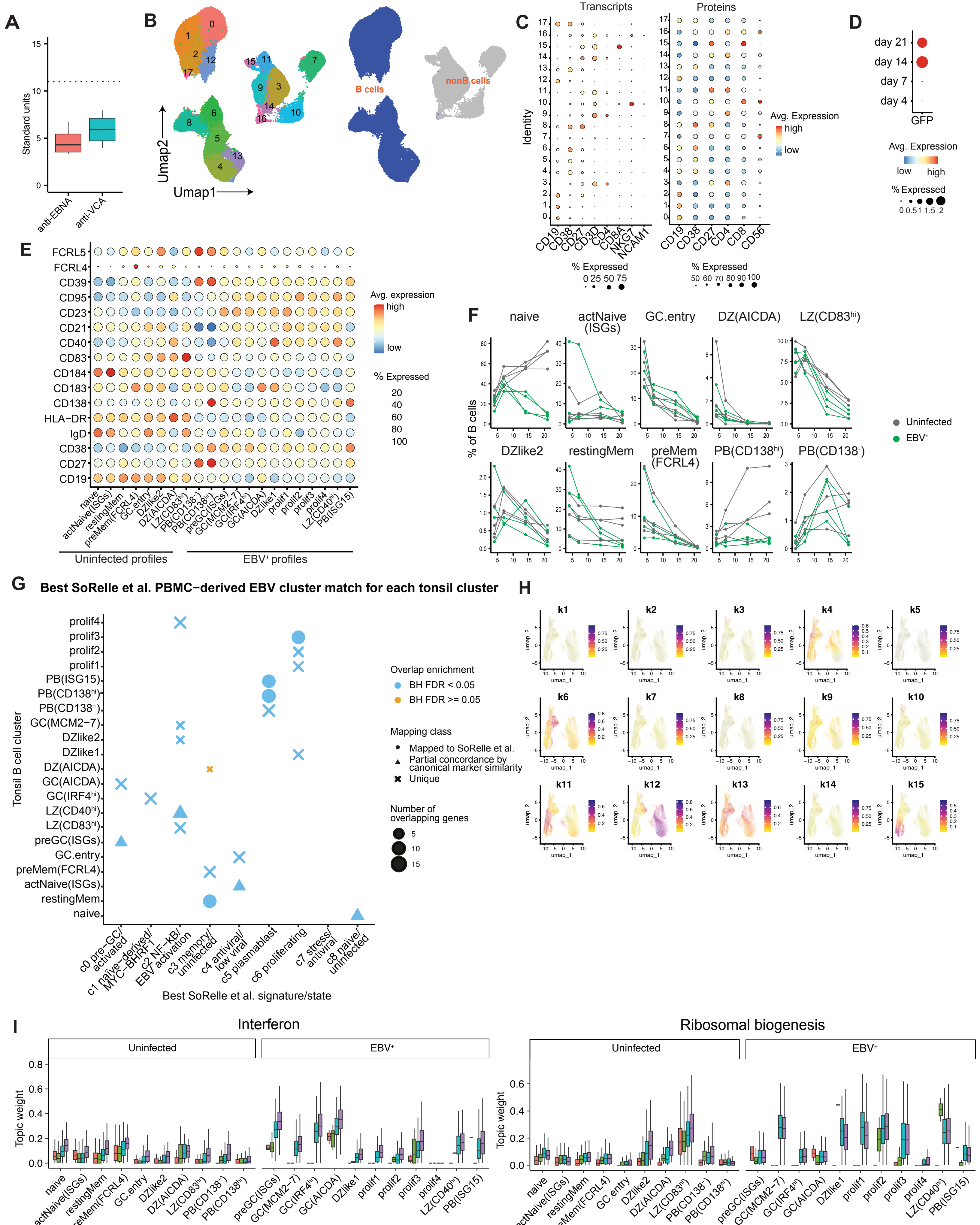

Figure S3: *See next page for figure legend.*

Figure S3: Features of EBV+ B cells in primary infection. (A) anti-EBNA and anti-VCA antibodies in donors used in the scRNAseq experiment (n=4). Dashed line indicates the threshold for seropositivity. (B, C) UMAP and features of B and non-B cells sorted from uninfected and EBV-infected organoids (89,444 cells, all donors, timepoints and stimulation conditions combined, n=4, 1 experiment). (D) Detection of GFP transcripts in infected organoids across time. (E) Bubble plot of cell surface markers used to identify B cell clusters. Bubble size indicates percentage of cells expressing the marker and color indicates magnitude of expression. (F) B cell cluster frequencies in EBV-infected and uninfected organoids from day 4 to day 21 (n=4 per group). None of the B cell cluster frequencies were significantly different. (G) Transcriptomic prolife comparison between the B cells derived from tonsil organoids and the PBMC-derived B cells described in SoRelle et al in response to EBV. Cluster comparisons defined as “mapped” had 10 or greater overlapping genes. (H) Feature plot showing the distribution of topics identified in EBV+ and bystander B cells. (I) Interferon and ribosomal biogenesis topic weights in B cell clusters in the EBV-infected organoids. Boxplots indicate the median value, with hinges denoting the first and third quartiles and whiskers denoting the highest and lowest value within 1.5 times the interquartile range of the hinges. Mann Whitney U tests followed by multiple hypothesis correction using the Benjamini & Hochberg (BH) method were used to calculate p values.

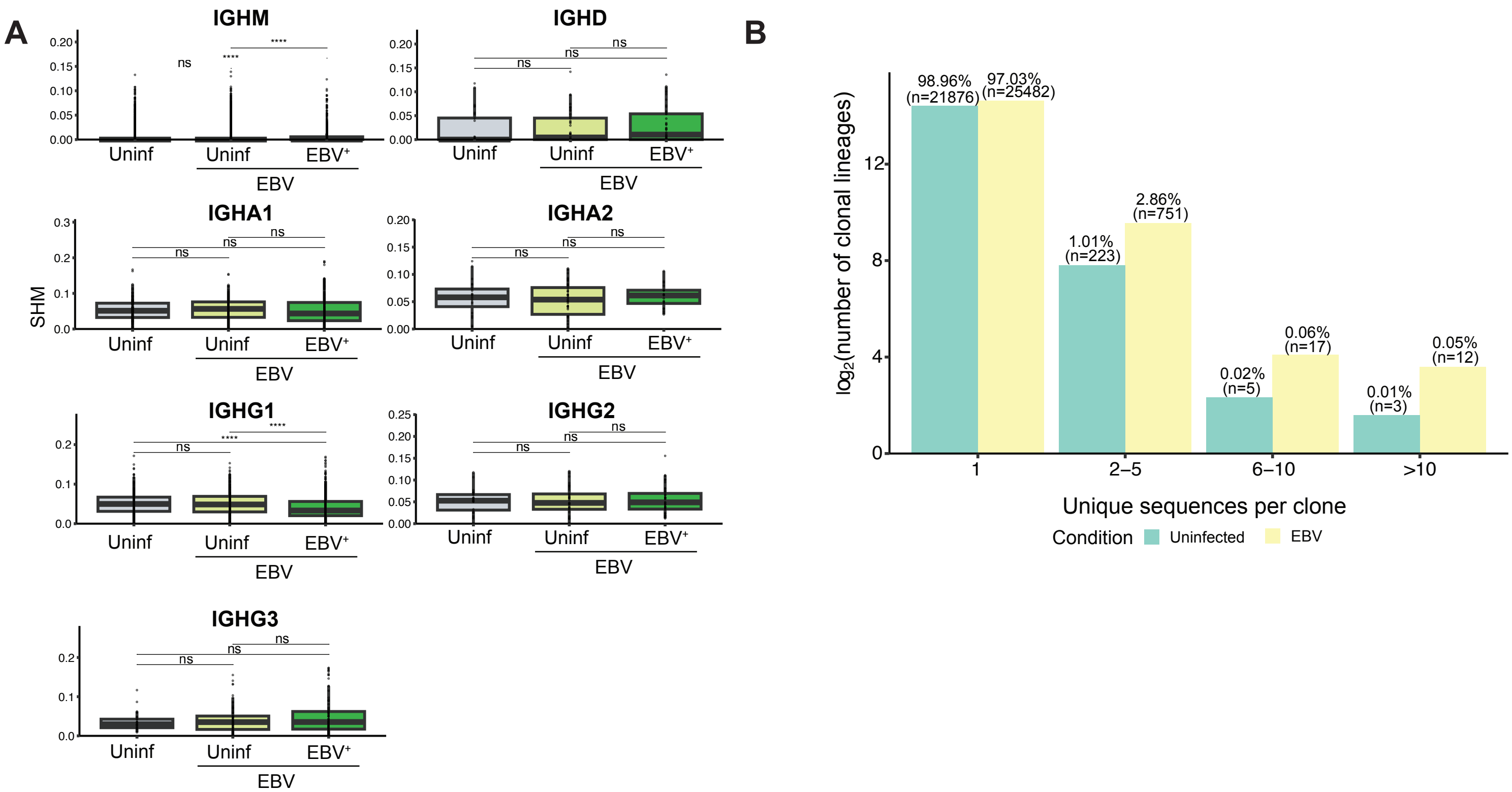

Figure S4. Additional B cell clonal features in EBV-infected tonsil organoids. (A) SHM frequency among immunoglobulin isotypes in uninfected, EBV-uninfected and EBV+ B cells on day 14. (B) Clone counts representing unique sequences in uninfected and EBV-infected organoids. Mann-Whitney U tests (two-sided) followed by false discovery rate correction by Benjamini & Hochberg method were used to calculate significance values (*p< 0.05, **p< 0.01, ***p< 0.001, ****p< 0.0001). SHM, Somatic hypermutation; ns, not significant (p ≥ 0.05).

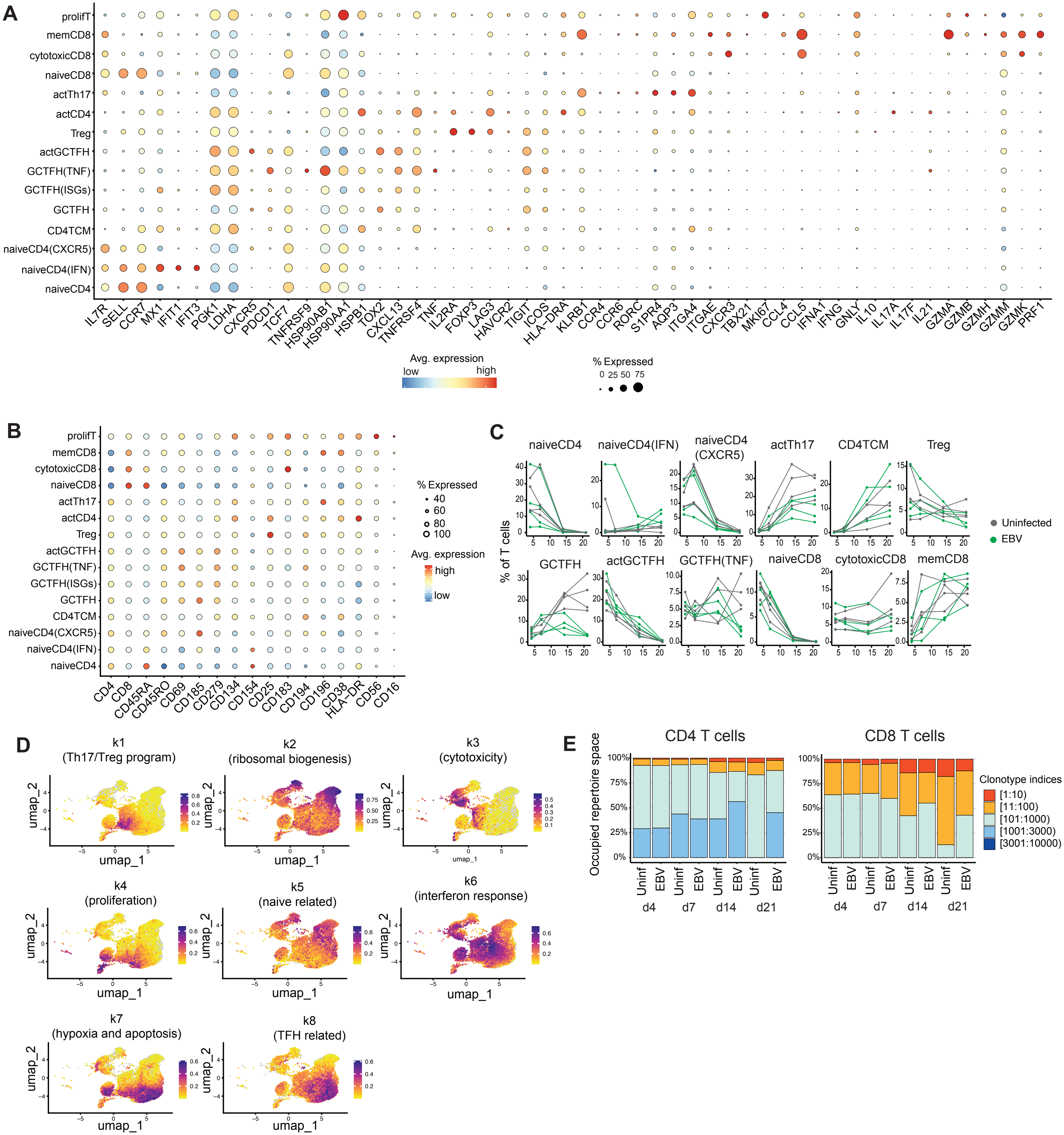

Figure S5: Additional features of T cell response in tonsil organoids during primary EBV infection. Bubble plots of defining (A) transcripts and (B) cell surface proteins in T cell subsets in primary EBV infection in tonsil organoids (all donors, timepoints and stimulation conditions aggregated, n=4, 1 experiment). Bubble size indicates percentage of cells expressing the transcript/marker and color indicates magnitude of expression. (C) Frequencies of CD4 T cell clusters in uninfected (gray) and EBV-infected (green) organoids during primary EBV infection (n=4 per group). No T cell cluster frequencies were significantly different between uninfected and EBV-infected organoids. (D) Feature plot showing the distribution of topics (gene expression program) in T cells (all timepoints, stimulation and donors aggregated). (E) Bar graph representing top clonal proportions of CD4 and CD8 T cells in uninfected and EBV-infected organoids on days 4, 7, 14 and 21 post infection. Mann Whitney U tests followed by multiple hypothesis correction using the Benjamini & Hochberg method were used to calculate p values.

Table S1: Tonsil, spleen and peripheral blood donor demographic information.

ID	Tissue type	Age (years)	Sex	Race	Source
1	Tonsil + Blood	13	F	White	CHOC
2	Tonsil + Blood	9	M	White	CHOC
3	Tonsil + Blood	6	M	Other	CHOC
4	Tonsil + Blood	3	M	White	CHOC
5	Tonsil + Blood	5	M	Asian	CHOC
6	Tonsil + Blood	6	F	Asian	CHOC
7	Tonsil + Blood	15	M	Other	CHOC
8	Tonsil + Blood	6	M	Asian	CHOC
9	Tonsil + Blood	4	M	White	CHOC
10	Tonsil + Blood	9	M	white	CHOC
11	Tonsil + Blood	4	M	White	CHOC
12	Tonsil + Blood	8	M	White	CHOC
13	Tonsil + Blood	12	M	Asian	CHOC
14	Tonsil + Blood	9	M	White	CHOC
15	Tonsil + Blood	2	F	Other	CHOC
16	Tonsil + Blood	15	M	White	CHOC
17	Tonsil + Blood	2	F	White	CHOC
18	Tonsil + Blood	5	M	White	CHOC
19	Tonsil + Blood	26	F	White	UCIMC
20	Tonsil	8	M	Black or African American	CHTN
21	Tonsil	16	F	White	CHTN
22	Tonsil	16	F	White	CHTN
23	Tonsil	5	F	White	CHTN
24	Tonsil	5	F	White	CHTN
25	Tonsil	3	M	White	CHTN
26	Tonsil	2	M	White	CHTN
27	Tonsil	11	M	White	CHTN
28	Tonsil	9	M	Black or African American	CHTN
29	Tonsil	2	F	White	CHTN
30	Tonsil	14	M	White	CHTN
31	Tonsil	11	F	White	CHTN
32	Tonsil	12	F	White	CHTN
33	Tonsil	3	M	Black or African American	CHTN
34	Tonsil	2	F	Black or African American	CHTN
35	Tonsil	11	F	White	CHTN
36	Tonsil	10	F	Black or African American	CHTN
37	Tonsil	8	F	Black or African American	CHTN
38	Tonsil	2	F	White	CHTN
39	Tonsil	10	F	White	CHTN
40	Tonsil	2	M	White	CHTN
41	Tonsil	9	F	Black or African American	CHTN
42	Tonsil	12	M	White	CHTN
43	Tonsil	14	F	White	CHTN
44	Tonsil	2	M	White	CHTN
45	Tonsil	3	F	White	CHTN
46	Tonsil	8	M	White	CHTN
47	Tonsil	5	M	White	CHTN
48	Tonsil	8	F	White	CHTN
49	Tonsil	13	F	White	CHTN
50	Tonsil	13	M	White	CHTN
51	Tonsil	17	F	Black or African American	CHTN

52	Tonsil	8	M	White	CHTN
53	Tonsil	3	F	White	CHTN
54	Tonsil	2	M	White	CHTN
55	Tonsil	8	F	White	CHTN
56	Tonsil	6	M	White	CHTN
57	Tonsil	2	F	White	CHTN
58	Tonsil	3	M	White	CHTN
59	Tonsil	4	M	White	CHTN
60	Tonsil	2	M	White	CHTN
61	Tonsil	7	F	White	CHTN
62	Tonsil	2	M	White	CHTN
63	Blood	28	F	White	ICTS
64	Blood	37	M	White	ICTS
65	Blood	53	M	White	ICTS
66	Blood	32	M	White	ICTS
67	Blood	30	M	Black	ICTS
68	Spleen	15	M	No data	CHOC
69	Spleen	77	F	More than one	UCIMC
70	Spleen	62	M	White	UCIMC
71	Spleen	75	M	White	UCIMC
72	Tonsil+Blood	6	M	African American	WUSTL
73	Tonsil+Blood	6	M	African American	WUSTL
74	Tonsil+Blood	19	F	Black or African American	UCIMC
75	Tonsil+Blood	19	M	White	UCIMC
76	Tonsil+Blood	11	F	White	WUSTL
77	Tonsil+Blood	4	F	Other	WUSTL
78	Tonsil+Blood	6	M	White	WUSTL
79	Tonsil+Blood	3	F	White	WUSTL
80	Tonsil+Blood	21	M	No data	UCIMC
81	Tonsil+Blood	16	M	More than one	CHOC
82	Tonsil+Blood	11	F	White	CHOC
83	Tonsil+Blood	17	M	White	CHOC
84	Tonsil+Blood	23	F	White	UCIMC

Supporting Information: Detailed Methods

EBV organoid infection dose selection

The optimal dose of the GFP-expressing EBV was established based on the ability to detect EBV-infected tonsil B cells by flow cytometry. A MOI of 0.000005, 0.00005 and 0.0005 of the GFP-expressing EBV (Akata) were used to infect the organoids and cells were harvested after 2 hours, on days 2, 5, 7, 14 post infection and subjected to flow cytometry analysis for detecting GFP^+ EBV^+ B cells. MOI of 0.0005 GFP-expressing EBV was found to be sufficient for detecting EBV^+ B cells in organoids by day 14. For infecting splenocytes and PBMCs the same dose of EBV was used. The cells were incubated at 37°C, 5% CO_2 and 1000uL or 100µL of fresh organoid media was added every 2-3 days or every other day by replacing approximately 500uL or 100µL media per well for the transwell or the 96 well plates respectively.

Cell depletion and organoid preparation

For naive, memory, or preGC B cell depletion experiments the *ex vivo* tonsil samples were stained with naive, memory, or preGC B cell markers and subjected to FACS sorter for a 2-way sort (naive, memory, or preGC B cells in one FACS tube and everything else in another FACS tube). Following depletion of naive, memory, or preGC B cells, organoids were prepared from each of the depleted conditions. For the depleted controls, the naive, memory, or preGC B cells were added back to other non-sorted cells in their native proportions and organoids were prepared. For depleting total CD4 or CD8 T cells we used MACS columns. In short, the cryopreserved tonsil cells are stained with CD4 or CD8 MACS beads following manufacturer's instructions. LS columns were used to separate the CD4 or CD8 T cells using a positive selection method. After separation, cells were enumerated and organoids prepared from the flow though portions (CD4 or CD8 depleted). For non-depleted controls, CD4 or CD8 T cells were added back to the flow though in their native proportions. The depleted and non-depleted organoids were maintained according to the above-mentioned method.

Flow cytometry

For cell surface characterization of the immune cells in the *ex vivo* tonsil tissues and organoids, cells were stained with separate B cell and T cell or a combined panel panel following established staining protocols. Briefly, thawed cells or harvested organoids were washed with FACS buffer (PBS + 0.1% BSA, 0.05% sodium azide and 2mM EDTA) and stained with live-dead staining dye (Zombie aqua) in PBS for 10 minutes on ice. Then cells were washed with FACS buffer and stained with a cocktail of antibodies (Biolegend) in FACS buffer for 30 minutes on ice. The stained cells were washed twice with the FACS buffer and analyzed using either Novocyte Quanteon (ACEA) instrument or the Northern lights or Aurora spectral cytometer and the acquired data was analyzed using FlowJo software (v10.8.1). Flow cytometry data from this study were deposited in Zenodo and are available under DOI https://doi.org/10.5281/zenodo.21478023.

Serotyping for EBV

For testing prior exposure to EBV, serum samples were tested for anti-VCA IgG and anti-EBNA1 IgG according to manufacturer's (Abcam) guideline. In brief, the serum samples were

diluted 1:100 with IgG sample diluent. 100uL of the diluted samples and standards (IgG positive control, IgG negative control, IgG cut-off control) were added in duplicates in the precoated (VCA or EBNA1) 96 well plate. The plates were sealed and incubated at 37C for an hour. After that the contents of the well were aspirated, washed three times with a wash buffer and Add 100 μL EBV anti-IgG HRP Conjugate were added followed by 30 minutes incubation at room temperature. After washing three times 100uL of TMB substrate was added in each well and incubated for 15 minutes. 100uL stop solution was added and the absorbance was measured at 450 nm. Sero-status for EBV was calculated based on standard units (SU; mean sample absorbancex10/cut-off) i.e. SU> 11 positive, SU<9 negative.

Cell sorting and single cell RNA sequencing

All scRNA sequencing data (GEO: GSE317492) have been deposited in the Gene Expression Omnibus database (https://www.ncbi.nlm.nih.gov/geo/). For scRNAseq kinetics analyses from EBV-infected or uninfected organoids from days 4, 7, 14, and 21 were harvested and cells were washed with FACS buffer to remove any residual antibodies/factors generated during culture. Samples were stained with a cocktail containing fluorescent-labeled antibodies DNA oligo-tagged antibodies and sample-specific hashtag antibodies (BioLegend). Samples were washed with FACS buffer and on days 4 and 7 total $CD45^+$ immune cells were sorted. We also sorted $CD45^+$ immune cells from *ex vivo* tonsil samples of the donors. On days 14 and 21 we sorted EBV^+ B cells (based on GFP expression), nonB cells from infected and uninfected organoids together and uninfected B cells from EBV-infected and uninfected organoids together. Sample hashing with hashtag oligos (HTOs) allowed us to pool several stimulation conditions into one tube. Pooled samples were resuspended in ice-cold PBS with 0.04% BSA in a final concentration of 2500 cells/μl. Single-cell suspensions were then immediately loaded on a 10X Genomics Chromium Controller with a loading target of 30,000 cells. Libraries were generated using the Chromium Next Gem Single Cell 5' Reagent Kit v2 (Dual Index) per the manufacturer's instructions, with additional steps for amplification of TotalSeq/HTO barcodes and V(D)J libraries (10X Genomics). Quality and quantity of libraries were measured on tapestation, qubit, and Bioanalyzer, and sequenced on Illumina NovaSeq 6000 with a sequencing target of 1500 million paired reads per cell for gene expression libraries, and 150 million paired reads per cell for TCR, and BCR libraries and 150 million paired reads per feature barcode libraries.

5′ gene expression analysis

Raw reads from gene expression, TCR, BCR, and HTO libraries (where applicable) were aligned and quantified using cellranger multi 8.0.1 against GRCh38-2024.-A which included the EBV genome (KC207813) and GFP transcript as extra chromosomes. VDJ libraries were aligned against refdata-cellranger-vdj-GRCh38-alts-ensembl-7.1.0. Gene expression matrices were loaded with Seurat v5.0.0, and EBV viral genes and GFP were isolated into a separate expression slot. TCR gene expression was aggregated (TRA, TRB, TRG, TRD) by gene family. HTO classification and demultiplexing were performed via the HTODemux function. A lower threshold of nFeature_RNA > 200 was applied across all samples. Upper thresholds for nFeature_RNA were adjusted based on sample type: a threshold of nFeature_RNA < 4000 was applied to baseline (Day 0) and non–B cell samples, while EBV-enriched B cell samples from Day 14 and

Day 21 were filtered with an upper threshold of nFeature_RNA < 6000. Additionally, a mitochondrial content threshold of <10% was applied to exclude cells with elevated mitochondrial gene expression.

Data normalization and variance stabilization were performed on the integrated object using NormalizeData and ScaleData functions in Seurat. Dimension reduction was performed using the RunPCA function and RunUMAP function was used to generate cell clustering at various resolutions. Cell types were broadly assigned as CD3D expressing T cells and CD19 expressing B cells, CD19- CD3- (nonBT) cells which were further subsetted for reclustering individually as described above. Gene and protein markers for each cluster were identified using FindMarkers function. For gene expression, we used Wilcoxon Rank Sum tests for differential marker detection using a $\log_2$ fold change cutoff of at least 0.25 and clusters were identified using a known catalog of markers defined for T and B cells from human tonsil. A similar approach was followed for protein expression, following normalization of antibody capture using centered logratio (CLR) transformation. A combination of both positive and negative protein markers from each cluster was used to aid annotations. Cluster-specific differential expression analysis across conditions was tested using the Wilcoxon rank-sum test with default settings in Seurat. Only statistically significant genes ($\log_2$ fold change cutoff of 0.4; adjusted p-value < 0.05) were included in downstream analyses.

To identify transcriptional programs in EBV-infected tonsil organoids in T and B cells, we performed topic modeling on scRNA-seq data using the R package fastTopics (v0.6-192)(1). Cells were subset from previously annotated Seurat objects which included T and B cell subclusters derived from samples collected at days 4, 7, 14, and 21 post-infections. Raw UMI counts were extracted from the RNA assay and filtered to remove genes with zero total counts across cells. Topic modeling was performed with the fit_topic_model function with default parameters. Topic-specific differentially expressed genes were determined with the de_analysis function and filtered to retain genes with a lfsr < 0.01. The top differentially expressed genes in each topic were used to infer the transcriptional program.

BCR analysis

BCR data was processed with Immcantation (https://immcantation.readthedocs.io/). Genes were first assigned with IgBLAST 1.21.0. Next, heavy V gene allele reassignment was performed with TIgGER 1.0.0. Prior to defining clonal families, hamming distances were calculated with SHazaM 1.1.2 distToNearest fucntion for cells with the same V gene, J gene, and junction length. Clonal families were then constructed with SCOPer 1.2.1 hierarchicalClones in single-cell mode. Germline sequences were inferred with Dowser 1.1.0 using sequences from the IMGT database (compiled November 2024). SHM was calculated with the SHazaM observedMutations function. Clonal lineage trees were constructed with heavy chain sequences with IgPhyML 1.1.5 and visualized with ggtree 3.6.0. Statistics were calculated with ggpubr 0.6.0.

TCR analysis

TCR sequence data were processed using Immunarch (https://immunarch.com/). Briefly, annotations generated by cellranger were filtered based on cells that passed all filters in the final UMAP. Analysis was performed on a cluster-, time-, stimulation and donor-specific manner. Data were first parsed on Immunarch using the *repLoad* function with a paired option, where only cells

with one productive alpha and one productive beta chain were retained for downstream analyses. Clonality (size, top clones, rare clones, and clonal homeostasis) was examined using the *repExplore* function. Diversity estimates (D50 and Chao) were measured using the *repDiversity* function.

Marker gene overlap analysis with published EBV-associated B cell states

For each transcriptionally defined cluster, the cluster best matched to SoRelle *et al*.(2) was selected based on the number of shared marker genes and the strength of marker overlap enrichment. Enrichment of marker overlap between each tonsil organoid cluster and each SoRelle *et al*. cluster was assessed using a one-sided Fisher's exact test, with p values adjusted across cluster comparisons using the Benjamini–Hochberg method. Clusters were classified as mapped to similar state if the best match had an adjusted p value <0.05 and contained >10 overlapping (20% overlap required) marker genes.

Immunofluorescence microscopy

Immunofluorescence microscopy was performed on formalin-fixed, paraffin-embedded (FFPE) sections derived from EBV-infected tonsil organoids. Permeable membrane inserts containing organoids were transferred to a new 12-well plate containing 1 mL cold RPMI medium per well and incubated at 4°C for 20 minutes. Thrombin (0.8 µL of a 5 U/µL stock; Sigma-Aldrich) was then added directly into each insert while keeping the samples on ice, followed by gentle shaking at approximately 150 rpm for 1 minute on ice. After thrombin addition, the plate was incubated again at 4°C for 20 minutes. Subsequently, 100 µL fibrinogen (10 mg/mL; Sigma-Aldrich) was added into each insert on ice, the plate was shaken gently at approximately 150 rpm for 1 minute on ice and then incubated at 37°C for at least 4 hours to allow fibrin gel formation. Following gel polymerization, the membrane was carefully removed from each insert using forceps to isolate the fibrin-embedded organoid gels. The gels were fixed with 4% paraformaldehyde in PBS for 24 hours at 4°C. Gels were then processed by routine FFPE procedures and sectioned for immunofluorescence staining.

FFPE sections were deparaffinized, rehydrated, and subjected to heat-induced antigen retrieval in ADCP2 buffer using a PT module (97°C, 20 min). After cooling and washing in distilled water, tissue sections were rinsed in TBS-T and blocked in 50% BBG blocking solution for 1 hour at room temperature before antibody staining. Primary antibodies were diluted in 0.25% casein and applied to the sections for overnight incubation at 4°C. The following primary antibodies and dilutions were used: anti-GFP (Aves Labs, GFP-1020), chicken IgY, 1:500; anti-CD3, rabbit monoclonal (Thermo, MA1-90582), 1:200; anti-CD20 (clone L26) conjugated to Alexa Fluor 647 (Thermo, 756-0202-94), mouse, 1:200; and anti-CD10, polyclonal goat antibody (Invitrogen, PA5-47075), 1:40. Following primary antibody incubation, slides were rinsed in 1× TBS-T for 5 minutes. For unconjugated primary antibodies, species-specific fluorescent secondary antibodies diluted in 0.25% casein were applied for 2 hours at room temperature as follows: donkey anti-chicken Alexa Fluor 488, 1:500; donkey anti-rabbit Alexa Fluor 568, 1:500; and donkey anti-goat DyLight 755, 1:250. After secondary antibody incubation, slides were rinsed in TBS-T for 5 minutes, and nuclei were counterstained with Hoechst (1:300 in buffer) followed by a final rinse in TBS-T. After final washes, sections were mounted with ProLong Gold antifade mounting medium and cover slipped.

Slides were imaged using an Akoya Biosciences multispectral fluorescence slide scanner ("Lego" model) equipped with a Sony IMX421 sCMOS camera and XCite multiband LED illumination at the Tissue Technologies Unit and Immunology Technologies Core, Center for Virology and Vaccine Research, Beth Israel Deaconess Medical Center. Images were acquired with a 10× objective (pixel size 0.499 μm) using six fluorescence channels (DAPI, Opal 520, Opal 620, Opal 690, Opal 780, and autofluorescence), exposure times ranging from 3 to 500 ms, 14-bit acquisition, 1×1 binning, gain set to 1, and Semrock multiband excitation/emission filters. QuPath 0.7.0 was used for visualization of Multispectral QPTIFF image files.

Methods References

1. P. Carbonetto, *et al.*, GoM DE: interpreting structure in sequence count data with differential expression analysis allowing for grades of membership. *Genome Biol.* **24**, 236 (2023).
2. E. D. SoRelle, *et al.*, Time-resolved transcriptomes reveal diverse B cell fate trajectories in the early response to Epstein-Barr virus infection. *Cell Rep.* **40**, 111286 (2022).
